# Flash Communication: Boron K‑edge XAS and TDDFT Studies of Covalent Metal–Ligand Bonding in Ni(C_2_B_9_H_11_)_2_

**DOI:** 10.1021/acs.organomet.5c00121

**Published:** 2025-06-06

**Authors:** Hannah M. Hansen, Palak Garg, Jacob J. Schuely, Mai Yer Yang, Omar K. Farha, Jason M. Keith, Scott R. Daly

**Affiliations:** † 4083 The University of Iowa, Department of Chemistry, E331 Chemistry Building, Iowa City, Iowa 52242, United States; ‡ 3270 Northwestern University, Department of Chemistry and International Institute for Nanotechnology, 2145 Sheridan Road, Evanston, Illinois 60208, United States; § 3719 Colgate University, Department of Chemistry, 13 Oak Drive, Hamilton, New York 13346, United States

## Abstract

Ligand K-edge X-ray absorption spectroscopy (XAS), a technique that can measure variations in covalent metal–ligand bonding, has rarely been used to assess covalency in complexes containing metal–boron bonds. Here we describe ligand K-edge XAS and TDDFT studies of the Ni dicarbollide complex Ni­(C_2_B_9_H_11_)_2_ (**1**) and the Ni-free salt (HNMe_3_)­(C_2_B_9_H_12_) (**L1**). The XAS spectrum for **1** reveals a pre-edge feature indicative of covalent Ni–B bonding, which is corroborated by time-dependent density functional theory (TDDFT) calculations and comparative analysis to **L1** and inner-shell electron energy loss spectroscopy (ISEELS) collected on the same Ni complex.

## Graphical abstract

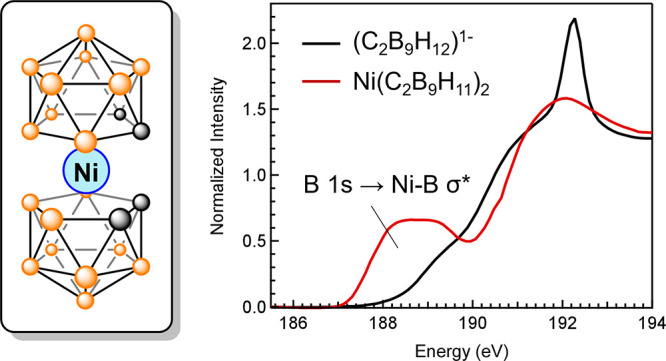

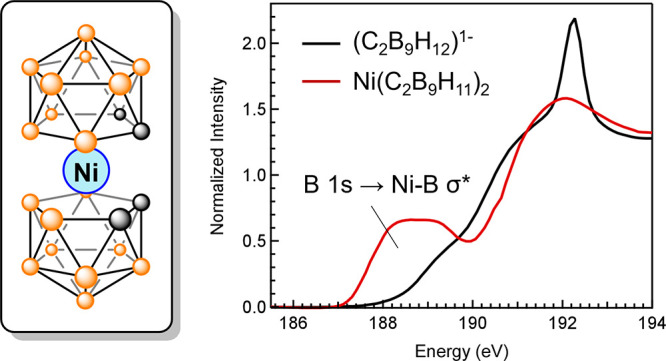

Assessing metal–ligand covalency, the degree of orbital mixing between metal and ligand atoms,^1^ is important to understanding the reactivity and electronic properties of metal complexes. Ligand K-edge X-ray absorption spectroscopy (XAS) is one of the few experimental methods available that can provide a direct measure of covalent metal–ligand bonding in metal complexes and materials. Ligand K-edge XAS was developed initially for analysis of metal–ligand bonds with Cl and S,^2,3^ but these efforts have since been extended to other ligand elements commonly used to form metal–ligand bonds like P,^4–14^ C,^15–20^ N,^21–25^ O,^26–30^ and F.^31^

One of the few elements that has yet to be investigated by ligand K-edge XAS is boron. Though B K-edge XAS has been used for many years to study minerals,^32^ intermetallic borides,^33^ and other extended solids,^34^ there appears to be few (if any) examples of this technique being used to investigate metal–boron bonding in molecular transition metal complexes. This absence is notable given that boron ligands like dicarbollides^35–38^ and borohydrides^39,40^ have been known since the mid-20th century. Moreover, boryl and borylene ligands have been used extensively in organometallic chemistry and catalysis,^41–50^ as have boratranes and related borane ligands that can form Z-type M→B bonds with electron rich metals.^51–53^

A complementary technique to XAS called inner-shell electron energy loss spectroscopy (ISEELS) has been used to assess B K-edge transitions in molecular complexes, including carboranes.^54,55^ ISEELS differs from XAS in that it relies on energy changes when electrons are inelastically scattered in response to inner shell excitations of light atom core electrons.^56,57^ However, despite its more extensive use in the analysis of boron containing molecules compared to XAS, ISEELS has also rarely been used to analyze complexes containing metal–boron bonds because this technique requires appreciably volatile complexes for analysis.^58^ In this context, B K-edge XAS offers an advantage in that it can be used to analyze nonvolatile samples.

Herein, we present B K-edge XAS studies of metal–boron bonds in the dicarbollide complex Ni­(C_2_B_9_H_11_)_2_ (**1**; Figure 1). This complex was first reported by Hawthorne and co-workers,^59–61^ and it was desirable for initial B K-edge XAS studies because of its relatively high symmetry, low-spin d^6^ electron configuration, and air stability. It is also a rare example of a stable Ni­(IV) complex, which highlights how dicarbollide ligands can stabilize metals in unusual redox states. Moreover, **1** is one of the few metal complexes that has been analyzed at the B K-edge using ISEELS. These ISEELS data for **1** were published in an online database^62,63^ by Hitchcock and co-workers and have yet to be described in the literature. We show here how they compare with the B K-edge XAS data.

**Figure 1:**
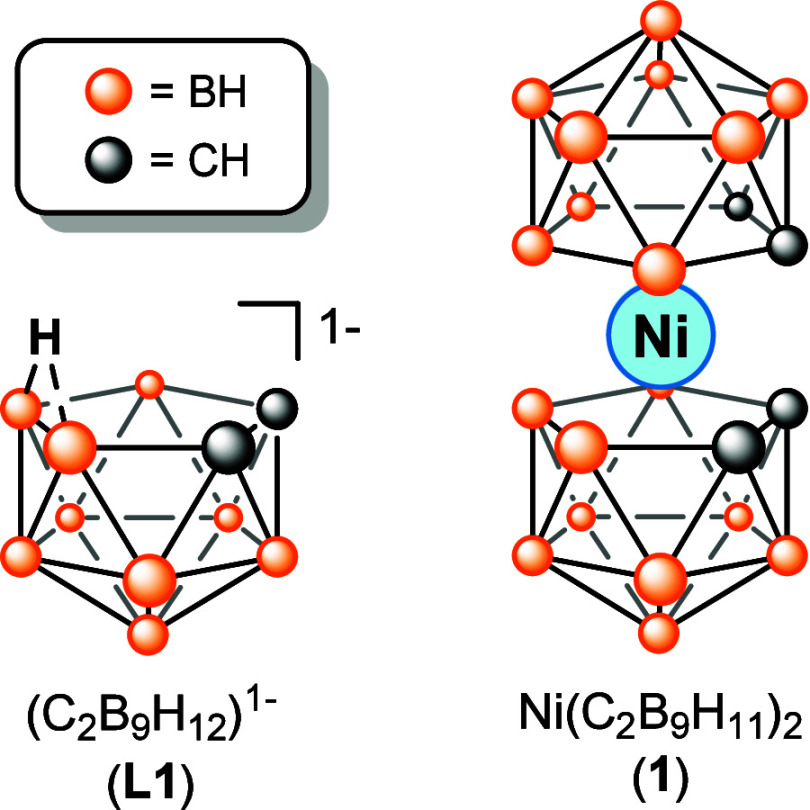
Structural comparison of **L1** and **1**.

B K-edge XAS data were collected at the Canadian Light Source (CLS) on the Variable Line Spacing Plane Grating Monochromator (VLS-PGM) beamline. The samples were ground into a powder and pressed into indium foil for measurements. All data were collected in fluorescence mode using a microchannel plate detector (see experimental details; ESI). Data were collected over a narrow energy window (184 – 210 eV) and the sample holder was moved between scans to minimize sample photodecomposition that became evident over time.

To help identify XAS features associated with Ni–B bonding, we first collected the B K-edge XAS spectrum of the Ni-free salt (HNMe_3_)­(C_2_B_9_H_12_) (**L1**; Figure 1), the ligand starting material used to prepare **1**. The B K-edge XAS spectrum of **L1** revealed two broad features centered at 189.2 and 190.8 eV, and these were more easily discernible in the first and second derivative traces (Figure 2 and Figure S3; ESI). A sharp absorption was also observed at higher energy at 192.3 eV on top of the rising edge. The line shape and resolution of this feature is reminiscent of those observed for boron oxides and nitrides,^32^ suggesting that it may be attributed to an impurity, potentially due to the photodecomposition described below.

**Figure 2:**
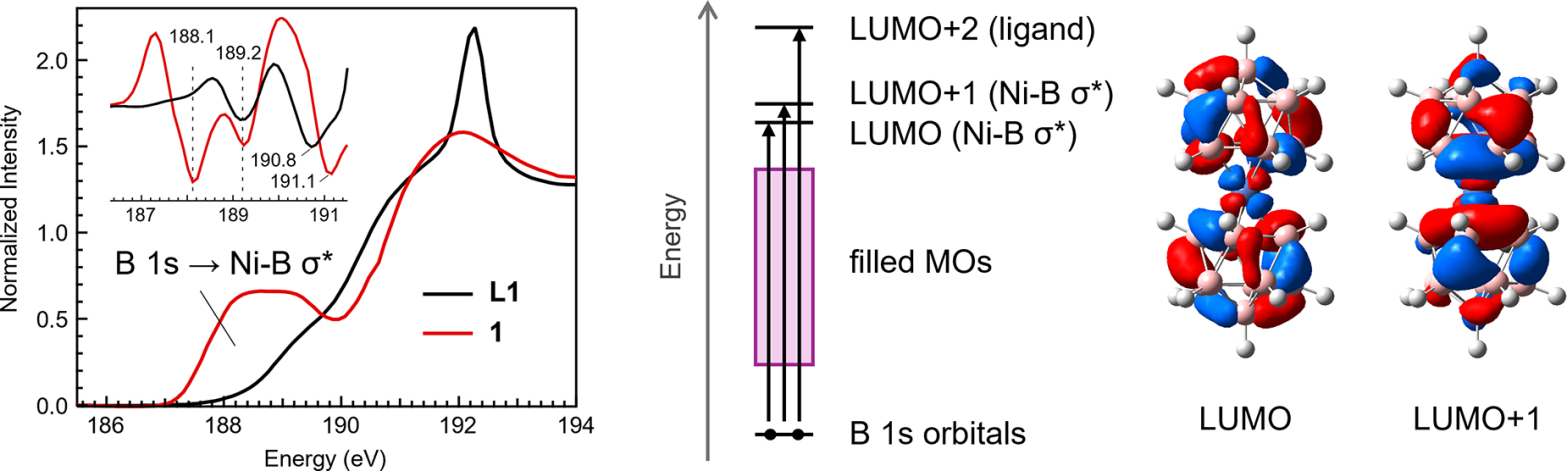
*Left* – Normalized B K-edge XAS spectra of **L1** (black trace) and **1** (red trace) with their second derivative traces (inset). *Right* – qualitative MO diagram for **1** with Kohn–Sham orbitals of the LUMO and LUMO+1 from DFT calculations.

The B K-edge XAS spectrum of **1** revealed a broad and pronounced pre-edge feature not observed in the salt **L1** (Figure 2). The second derivative of the trace indicates the pre-edge feature is comprised of at least two absorptions centered at 188.1 and 189.2 eV, followed by a higher energy feature centered at 191.1 eV. The broad pre-edge feature in the B K-edge XAS spectrum of **1**, and the lack of such a feature in the spectrum of **L1**, suggests that it can be assigned to transitions associated with Ni–B bonds. This was indeed corroborated by density functional theory (DFT) calculations.

Dispersion-corrected DFT calculations (B3LYP-d3 ^64,65^ with LANL08­(f)^66–68^ and double-ζ 6–31G­(d,p) basis sets^69^) were performed on both **L1** and **1**. The calculated structures reproduce those observed by single-crystal X-ray diffraction (XRD). Notably, the structure of **1** has C_2_ point group symmetry with the C_2_ rotation axis situated between the staggered C–C bonds on the same side of opposing dicarbollide ligands. The calculations performed on **1** revealed the LUMO and LUMO+1 to be Ni–B σ* between the Ni 3d_*xz*_ and 3d_*yz*_, respectively, and out-of-phase orbitals delocalized over the dicarbollide ligands (Figure 2). The LUMO for **1** consists of 49% B 2p and 28% Ni 3d character, and the LUMO+1 is similar at 47% and 25% for B 2p and Ni 3d, respectively. The LUMO and LUMO+1 are relatively close in energy, separated by ca. 0.09 eV. Beyond the LUMO+1 at higher energy is a manifold of ligand-centered orbitals with minimal Ni character. The ligand-localized LUMO+2 is 3.9 eV higher in energy relative to the LUMO+1.

Time-dependent density functional theory (TDDFT) calculations were performed using the ground-state DFT outputs for **L1** and **1** to simulate the B K-edge XAS spectra and identify transitions responsible for the observed features (Figure 3). The simulated spectra reproduce the most salient features of the experimental data with a few exceptions that will be accounted for below. Of the 18 boron 1s orbitals in **1**, there are nine unique B 1s environments that give rise to a manifold of B 1s → Ni–B σ* transitions involving the LUMO and LUMO+1 that account for the pre-edge region in the B K-edge XAS spectrum of **1** at 188.1 eV. At higher energy are B 1s transitions to ligand-based orbitals delocalized over the dicarbollide framework. The pre-edge transitions are not observed in the absence of Ni for **L1**, but similar ligand-centered transitions at higher energy account for the absorptions observed above 190 eV.

**Figure 3:**
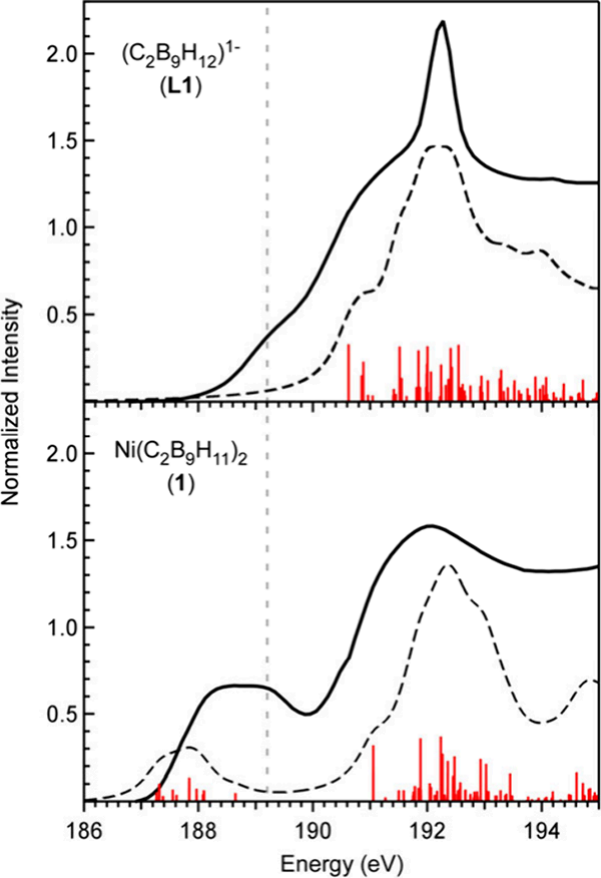
Comparison of experimental and simulated B K-edge XAS data for **L1** (*top*) and **1** (*bottom*). The experimental spectra (solid lines), simulated spectra (dashed lines), and calculated transitions (red bars) are shown. Calculated oscillator strengths were multiplied by a factor of 10 to bring them on scale with the experimental data. An energy shift of +9.0 eV was applied to the calculated spectra so that relative differences in calculated and experimental peak positions could be compared. The dashed vertical line in gray is to emphasize the pre-edge feature assigned to photodecomposition in both **L1** and **1**.

The TDDFT calculations do not account for the features observed at 189.2 eV in the B K-edge XAS spectra of **L1** and **1**; these features are absent in the simulated spectra for both complexes (emphasized in Figure 3 with the vertical dashed line), suggesting that they are not attributed to the parent complexes. Consistent with this assessment, we discovered that **L1** and **1** both undergo slow photodecomposition in the beam, which was evident when repeatedly collecting scans on the same sample position (Figures S4 and S5; ESI). Both complexes show the ingrowth of the features at 189.2 eV. Photodecomposition may also account for the sharp absorption in the spectrum of **L1** at 192.3 eV, which also appears to increase when scans are collected on the same spot (Figure S4).

Coincidentally, ISEELS studies reported previously may account for the identity of the decomposition product, at least for **1**. The ISEELS spectrum and structure of *nido*-7,8-dicarbaundecaborane­(11) (*nido*-7,8-C_2_B_9_H_11_), the neutral C_2_B_9_H_11_ form of the dicarbollide ligand in **1**, is shown in Figure 4.^54^ This spectrum was generated *in situ* by thermal decomposition of **1** to form *nido*-7,8-C_2_B_9_H_11_ and presumably elemental Ni. Relatedly, Hawthorne and co-workers reported a similar preparation of C_2_B_9_H_11_ from thermal decomposition of *commo*-3,3′-Si­(3,l,2-SiC_2_B_9_H_11_)_2_ and deposition of Si.^70^ The ISEELS spectrum of C_2_B_9_H_11_ revealed a pre-edge absorption at 189.1 eV, which is nearly identical to the energy of the photodecomposition product observed in the B K-edge XAS spectra of **1** and **L1**. Moreover, the low-resolution ISEELS spectrum of **1** reported in the Gas Phase Core Excitation Database shows a pre-edge feature at lower energy centered around 188 eV (Figure 4).^62,63^ The comparison of the ISEELS and TDDFT data to the B K-edge XAS spectrum of **1** suggests that the first pre-edge feature at 188.1 eV can be assigned to Ni–B σ* transitions and the second pre-edge feature at 189.2 eV can be attributed to photogenerated *nido*-7,8-C_2_B_9_H_11_.

**Figure 4:**
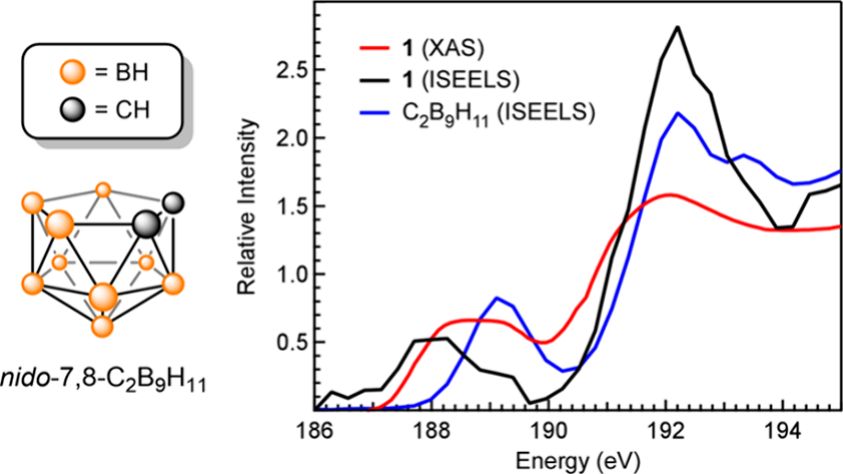
*Left* – Structure of *nido*-7,8-C_2_B_9_H_11_. *Right* – Comparison of the normalized B K-edge XAS spectrum of **1** (red) to ISEELS spectra of **1** (black) and *nido*-7,8-C_2_B_9_H_11_ (blue). The ISEELS data were obtained from the Gas Phase Core Excitation Database.^54,62,63^ The intensity of the ISEELS spectrum of *nido*-7,8-C_2_B_9_H_11_ is shown as oscillator strength per boron atom (10^–2^ eV^–1^), as reported previously.^54^ The intensity shown for the ISEELS spectrum of **1** is arbitrary and was modified to bring it on the same scale as the other spectra.

In summary, we have reported the B K-edge XAS spectra of the *nido*-carborane salt (HNMe_3_)­(C_2_B_9_H_12_) (**L1**) and the dicarbollide complex Ni­(C_2_B_9_H_11_)_2_ (**1**). These preliminary results demonstrate how metal–boron covalency may be assessed in coordination complexes using B K-edge XAS. The spectrum for **1** revealed a pre-edge feature at 188.1 eV that can be assigned to B 1s → Ni–B σ* transitions, which is consistent with (TD)­DFT calculations and ISEELS data collected on the same complex.

Though ligand K-edge XAS is typically associated with the analysis of covalent bonding in molecular complexes like **1**, we are careful to note that this is not the first time covalent M-B bonding has been identified using B K-edge XAS. For example, pre-edge features assigned to covalent Ni–B bonding were reported previously in the B K-edge XAS spectra of the intermetallic superconducting solids RENi_2_B_2_C, where RE = rare earth element.^71^ Interestingly, the energy of the 188.1 eV pre-edge feature reported here for **1** is effectively identical to those reported in RENi_2_B_2_C solids.^71^ This pre-edge feature is also similar to those assigned to covalent Mn–B bonding in the B K-edge XAS spectra of binary Mn borides.^72^ This suggests that B K-edge XAS may be used to relate the chemical bonding and electronic structure in intermetallic borides and materials to discrete coordination complexes that also contain M-B bonds. However, achieving these comparisons, especially with respect to quantifying pre-edge peak intensities, will require attenuation of beam-induced decomposition in coordination complexes like **1**, which is the focus of ongoing work.

## Supplementary Material



## Notes

Experimental and theoretical details, 1st and 2nd derivative XAS traces, tabulated DFT and TDDFT data (PDF)
