# Supplementary material for: Flash Communication: Boron K‑edge XAS and TDDFT Studies of Covalent Metal–Ligand Bonding in Ni(C2B9H11)2
Source: Organometallics. 2025 Jun 6;44(15):1624–9. doi: 10.1021/acs.organomet.5c00121 (PMC12344760; doi:10.1021/acs.organomet.5c00121)
Supplement: Supplementary file 1 [file om5c00121_si_001.pdf]

SUPPORTING INFORMATION

Flash Communication: Boron K-edge XAS and TDDFT Studies of Covalent Metal-Ligand Bonding in $Ni(C_2B_9H_{11})_2$

Hannah M. Hansen,[1] Palak Garg,[2] Jacob J. Schuely,[1] Mai Yer Yang,[1] Omar K. Farha,[2] Jason M. Keith,[3*] Scott R. Daly[1*]

[1]*The University of Iowa, Department of Chemistry, E331 Chemistry Building, Iowa City, IA 52242, United States*

[2]*Northwestern University, Department of Chemistry and International Institute for Nanotechnology, 2145 Sheridan Road, Evanston, Illinois 60208, United States*

[3]*Colgate University, Department of Chemistry, 13 Oak Drive, Hamilton, New York 13346, United States*

Corresponding emails: scott-daly@uiowa.edu; jkeith@colgate.edu

Table of Contents

Experimental Details S2

DFT and TDDFT Calculations S5

Supporting Information References S70

Experimental Details

B K-edge XAS. B K-edge XAS data were collected at the Canadian Light Source (CLS) in Saskatoon, Saskatchewan, Canada at the Variable Line Spacing Plane Grating Monochromator (VLS-PGM) beamline. Samples were loaded under Ar using an argon-purged glovebox attached to the sample chamber. Fluorescence yield (FLY) data were collected at room temperature using an MCP detector in a sample chamber maintained between 10^{-8} and 10^{-10} Torr. Incident light (I_0) current was measured in TopUp mode (I = 220 mA) using a Si photodiode ($XSUV_{100}$). Data were collected by scanning the energy from 210 eV to 184 eV in 0.1 eV steps with 1.0 s dwell times. In addition to limiting the scan window, photodecomposition was minimized by moving the sample position after each scan. The entrance and exit slit widths were set to 100 µm. Data were ratioed to the I_0 current, baseline subtracted by fitting a line to 184 to 187 eV, and normalized by setting the intensity of the 210 eV data point to 1.0. For final spectra, intensities of the respective samples were averaged and plotted where both **1** and **L1** had 17 measurements averaged each.

Except for indium foil, all materials used for XAS sample preparation and delivery to the beamline (Cu sample plates, double-sided Cu tape, mortars, pestles, and glass jars) were heated in an oven at 150 °C for a minimum of 1.5 h and allowed to cool under vacuum in the glovebox antechamber. Crystalline samples of $(HNMe_3)(C_2B_9H_{12})$ (**L1**) and $Ni(C_2B_9H_{11})_2$ (**1**) were ground using a mortar and pestle in an argon-filled glovebox maintained at <1.0 ppm O_2 and H_2O. The powders were pressed into indium foil mounted on Cu sample plates using double-sided copper tape (Figure S1). Once prepared, the sample plates were sealed in jars in the glovebox and transferred to the argon-purge box at the VLS-PGM beamline for loading. Indium foil was used as received from Alfa Aesar (99.99%). **L1** and **1** were prepared as described previously.[1]

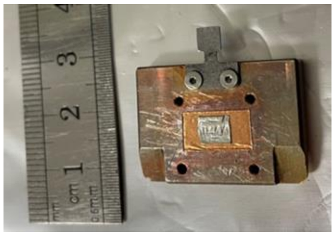

Figure S1. Sample plate setup showing In foil adhered to a Cu sample plate using double-sided Cu tape.

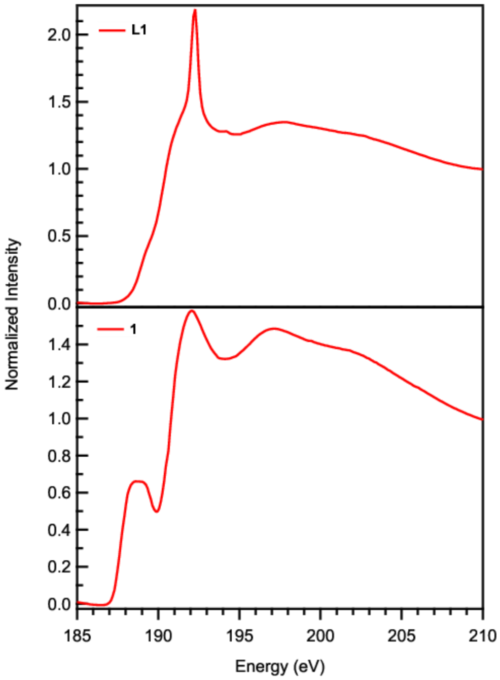

Figure S2. Normalized B K-edge XAS spectra for $(HNMe_3)(C_2B_9H_{12})$ (**L1**) and $Ni(C_2B_9H_{11})_2$ (**1**).

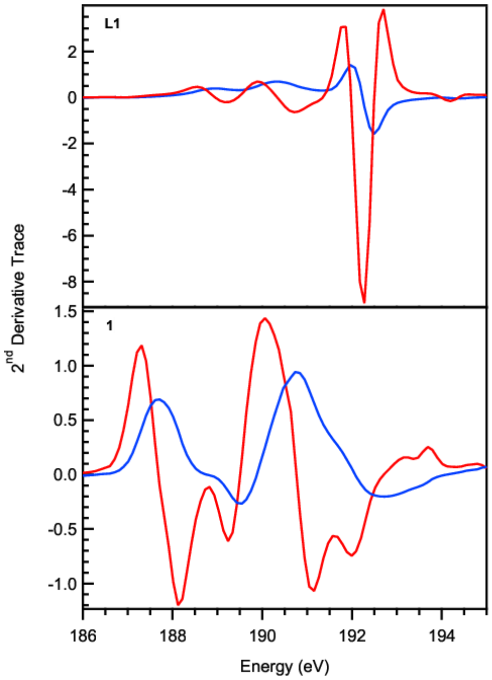

Figure S3. First (blue) and second (red) derivatives traces for normalized B K-edge XAS spectra for $(HNMe_3)(C_2B_9H_{12})$ (**L1**) and $Ni(C_2B_9H_{11})_2$ (**1**).

.

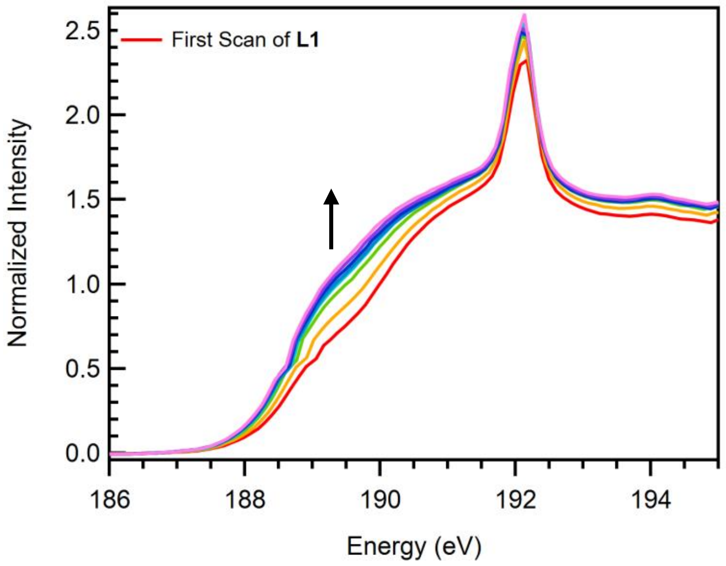

Figure S4. Repeat scans of $(HNMe_3)(C_2B_9H_{12})$ (**L1**) at same position.

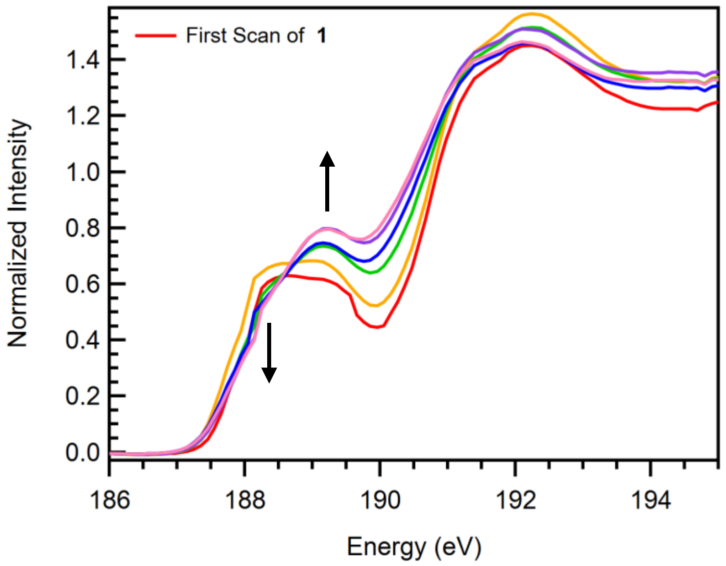

Figure S5. Repeat scans of $Ni(C_2B_9H_{11})_2$ (**1**) at same position.

DFT and TDDFT Calculations

Calculations were performed as described previously with density functional theory (DFT) as implemented by the Gaussian 09 computational chemistry suite.[2] Starting geometries were informed by the crystal structures and all structures were fully optimized. Initial optimizations were performed at the B3LYP-d3 level.[3] Ni was modeled with the effective core potential and uncontracted basis set of Hay and Wadt augmented with a set of f-orbital polarization functions collectively known as LANL08(f).[4] All other atoms were modeled with Pople's split-valence double-z 6-31G(d,p) plus polarization basis set.[5] The existence of the correct number of imaginary frequencies was determined through evaluation of the analytic hessian, which along with the potential energy and gradient information was used to generate the zero-point energy as well as enthalpy and free energy corrections to the internal energy.

Time-dependent density functional theory (TDDFT) calculations were combined with information on the charges, spin densities and relative orbital contributions from Mulliken population analysis[6] of the individual molecular orbitals from the ground state structures in order to simulate and interpret the XAS.

Table S1. Calculated orbital energies and atomic compositions for **L1**.

Assignment	MO #	Hartrees	eV	B p	B s	C p	C s
	38	0.20983	5.71	27%	4%	4%	1%
	37	0.1993	5.42	82%	0%	9%	0%
LUMO	36	0.17911	4.87	63%	2%	16%	1%
HOMO	35	-0.06783	-1.85	74%	2%	16%	1%
B 1s	11	-6.54984	-178.23	0%	100%	0%	0%
B 1s	10	-6.55104	-178.26	0%	100%	0%	0%
B 1s	9	-6.55593	-178.40	0%	100%	0%	0%
B 1s	8	-6.55615	-178.40	0%	100%	0%	0%
B 1s	7	-6.55687	-178.42	0%	100%	0%	0%
B 1s	6	-6.57098	-178.81	0%	100%	0%	0%
B 1s	5	-6.58028	-179.06	0%	100%	0%	0%
B 1s	4	-6.58048	-179.06	0%	100%	0%	0%
B 1s	3	-6.59462	-179.45	0%	100%	0%	0%

*Values of 0% correspond to atomic compositions $< 0.5\%$.

Table S2. Calculated orbital energies and atomic compositions for **1**.

Assignment	MO #	Hartrees	eV	Ni d	Ni p	Ni s	B p	B s	C p	C s
	80	-0.01011	-0.28	3%	0%	0%	69%	0%	22%	0%
	79	-0.15452	-4.20	25%	0%	0%	47%	2%	21%	2%
LUMO	78	-0.15778	-4.29	28%	0%	2%	49%	2%	3.6%	0%
HOMO	77	-0.28441	-7.74	0%	26%	0%	59%	0%	4%	0%
B 1s	22	-6.74144	-183.44	0%	0%	0%	0%	100%	0%	0%
B 1s	5	-6.79289	-184.84	0%	0%	0%	0%	100%	0%	0%

*Values of 0% correspond to atomic compositions $< 0.5\%$.

Table S3. Tabulated TDDFT data and transitions from B 1s orbitals for **L1**. An energy shift of +9.0 eV was applied to the calculated spectra so that relative differences in calculated and experimental peak positions could be compared.

Excited State	Calculated Energy (eV)	Shifted Energy (eV)	Oscillator Strength (f-value)	To Orbital	Coefficient
1	178.2182	187.2182	0.0016	78	0.21996
1	178.2182	187.2182	0.0016	78	0.66895
2	178.2186	187.2186	0.0007	78	0.66894
2	178.2186	187.2186	0.0007	78	-0.21995
3	178.2243	187.2243	0.0053	78	0.15947
3	178.2243	187.2243	0.0053	78	0.68102
4	178.2246	187.2246	0.0025	78	0.681
4	178.2246	187.2246	0.0025	78	-0.15926
5	178.2737	187.2737	0.0098	78	-0.16928
5	178.2737	187.2737	0.0098	78	0.68035
6	178.274	187.274	0.0102	78	0.68042
6	178.274	187.274	0.0102	78	0.16945
7	178.3372	187.3372	0.0032	79	-0.35131
7	178.3372	187.3372	0.0032	79	0.60913
8	178.3373	187.3373	0.0003	79	0.60913
8	178.3373	187.3373	0.0003	79	0.35133
9	178.3421	187.3421	0.003	79	0.5335
9	178.3421	187.3421	0.003	79	-0.45944
10	178.3422	187.3422	0.0008	79	0.45944
10	178.3422	187.3422	0.0008	79	0.53352
11	178.5078	187.5078	0.0023	79	0.70583
12	178.5089	187.5089	0.0069	79	0.70584
13	178.5118	187.5118	0.0031	78	0.70568
14	178.5122	187.5122	0.0031	78	0.70568
15	178.576	187.576	0.0038	79	0.70533
16	178.5761	187.5761	0.0037	79	0.70533
17	178.7998	187.7998	0.0021	78	0.56817
17	178.7998	187.7998	0.0021	78	0.2054
17	178.7998	187.7998	0.0021	79	0.33862
18	178.8002	187.8002	0.008	78	0.45501
18	178.8002	187.8002	0.008	79	-0.16656
18	178.8002	187.8002	0.008	79	-0.29051
18	178.8002	187.8002	0.008	78	-0.41416
19	178.8004	187.8004	0.0137	78	0.28923
19	178.8004	187.8004	0.0137	79	0.14454
19	178.8004	187.8004	0.0137	78	0.2164
19	178.8004	187.8004	0.0137	79	0.27102
19	178.8004	187.8004	0.0137	78	-0.35371
19	178.8004	187.8004	0.0137	79	-0.14335

19	178.8004	187.8004	0.0137	78	0.35436
20	178.8008	187.8008	0.0113	78	0.28892
20	178.8008	187.8008	0.0113	79	-0.3022
20	178.8008	187.8008	0.0113	79	0.10708
20	178.8008	187.8008	0.0113	78	0.46017
20	178.8008	187.8008	0.0113	79	-0.1055
20	178.8008	187.8008	0.0113	78	0.27866
21	178.9227	187.9227	0	78	0.11213
21	178.9227	187.9227	0	78	-0.31928
21	178.9227	187.9227	0	79	0.61078
22	178.9234	187.9234	0.0023	78	0.3209
22	178.9234	187.9234	0.0023	79	0.10676
22	178.9234	187.9234	0.0023	79	0.60318
23	178.9239	187.9239	0.0073	79	0.31072
23	178.9239	187.9239	0.0073	79	0.53048
23	178.9239	187.9239	0.0073	78	0.22657
23	178.9239	187.9239	0.0073	78	-0.22867
24	178.9239	187.9239	0.0038	79	0.52112
24	178.9239	187.9239	0.0038	79	-0.31722
24	178.9239	187.9239	0.0038	78	0.24186
24	178.9239	187.9239	0.0038	78	0.23745
25	179.0394	188.0394	0.0041	79	0.18162
25	179.0394	188.0394	0.0041	78	0.66321
26	179.0396	188.0396	0.001	79	-0.16288
26	179.0396	188.0396	0.001	78	0.65998
26	179.0396	188.0396	0.001	79	0.12594
27	179.0403	188.0403	0.0008	78	0.64899
27	179.0403	188.0403	0.0008	79	0.12867
27	179.0403	188.0403	0.0008	79	0.2318
28	179.0407	188.0407	0.0009	78	0.64925
28	179.0407	188.0407	0.0009	79	-0.14561
28	179.0407	188.0407	0.0009	79	-0.21081
29	179.0531	188.0531	0.001	78	-0.23618
29	179.0531	188.0531	0.001	79	0.10486
29	179.0531	188.0531	0.001	78	0.11959
29	179.0531	188.0531	0.001	79	0.64026
30	179.0537	188.0537	0.001	78	0.23321
30	179.0537	188.0537	0.001	78	-0.11868
30	179.0537	188.0537	0.001	79	0.64354
30	179.0537	188.0537	0.001	79	-0.105
31	179.0546	188.0546	0.0065	78	-0.13846
31	179.0546	188.0546	0.0065	79	0.65907
31	179.0546	188.0546	0.0065	78	-0.18903
32	179.055	188.055	0.0058	78	0.12759

32	179.055	188.055	0.0058	79	0.66294
32	179.055	188.055	0.0058	78	0.1877
33	179.6062	188.6062	0.0049	78	0.70563
34	179.607	188.607	0.004	78	0.70563
35	179.7314	188.7314	0.0003	79	0.70615
36	179.7319	188.7319	0.0002	79	0.70615
37	182.013	191.013	0.032	80	0.4645
37	182.013	191.013	0.032	83	0.23654
37	182.013	191.013	0.032	84	-0.22194
37	182.013	191.013	0.032	80	-0.39465
38	182.0133	191.0133	0.0162	80	0.39451
38	182.0133	191.0133	0.0162	80	0.46438
38	182.0133	191.0133	0.0162	83	-0.237
38	182.0133	191.0133	0.0162	84	0.22219
39	182.0147	191.0147	0.002	80	0.56004
39	182.0147	191.0147	0.002	83	0.23148
39	182.0147	191.0147	0.002	84	-0.21725
39	182.0147	191.0147	0.002	80	-0.2389
39	182.0147	191.0147	0.002	83	-0.10527
40	182.015	191.015	0.0021	80	0.23876
40	182.015	191.015	0.0021	83	-0.10545
40	182.015	191.015	0.0021	80	0.55992
40	182.015	191.015	0.0021	83	-0.23201
40	182.015	191.015	0.0021	84	0.21754
41	182.2261	191.2261	0.0023	80	0.60551
41	182.2261	191.2261	0.0023	83	0.28241
41	182.2261	191.2261	0.0023	84	-0.22024
42	182.2263	191.2263	0.0023	80	0.60518
42	182.2263	191.2263	0.0023	83	-0.28294
42	182.2263	191.2263	0.0023	84	0.22048
43	182.4593	191.4593	0.0031	80	0.6362
43	182.4593	191.4593	0.0031	83	0.21159
43	182.4593	191.4593	0.0031	84	-0.16851
44	182.4605	191.4605	0.0064	80	0.63593
44	182.4605	191.4605	0.0064	83	-0.21211
44	182.4605	191.4605	0.0064	84	0.16866
45	182.5449	191.5449	0.0065	82	0.24658
45	182.5449	191.5449	0.0065	85	-0.13392
45	182.5449	191.5449	0.0065	87	-0.13776
45	182.5449	191.5449	0.0065	82	0.5154
45	182.5449	191.5449	0.0065	85	-0.27946
45	182.5449	191.5449	0.0065	87	-0.18939
46	182.5452	191.5452	0.0045	82	0.51562
46	182.5452	191.5452	0.0045	85	-0.27898

46	182.5452	191.5452	0.0045	87	0.18938
46	182.5452	191.5452	0.0045	82	-0.24694
46	182.5452	191.5452	0.0045	85	0.13339
46	182.5452	191.5452	0.0045	87	-0.13791
47	182.5524	191.5524	0.001	82	0.18054
47	182.5524	191.5524	0.001	87	-0.1129
47	182.5524	191.5524	0.001	82	0.54641
47	182.5524	191.5524	0.001	85	-0.28885
47	182.5524	191.5524	0.001	87	-0.20156
48	182.5527	191.5527	0.0007	82	0.54673
48	182.5527	191.5527	0.0007	85	-0.28822
48	182.5527	191.5527	0.0007	87	0.2016
48	182.5527	191.5527	0.0007	82	-0.18074
48	182.5527	191.5527	0.0007	87	-0.11295
49	182.6835	191.6835	0	81	-0.33636
49	182.6835	191.6835	0	82	-0.11306
49	182.6835	191.6835	0	85	-0.28199
49	182.6835	191.6835	0	81	0.51236
50	182.6836	191.6836	0	81	0.51131
50	182.6836	191.6836	0	81	0.3359
50	182.6836	191.6836	0	82	-0.11439
50	182.6836	191.6836	0	85	-0.28364
51	182.6893	191.6893	0.0003	81	0.46586
51	182.6893	191.6893	0.0003	82	0.10678
51	182.6893	191.6893	0.0003	85	0.27773
51	182.6893	191.6893	0.0003	81	-0.40186
52	182.6893	191.6893	0.001	81	0.40115
52	182.6893	191.6893	0.001	81	0.46545
52	182.6893	191.6893	0.001	82	-0.10778
52	182.6893	191.6893	0.001	85	-0.27891
53	182.7104	191.7104	0.0034	81	0.15751
53	182.7104	191.7104	0.0034	82	0.17056
53	182.7104	191.7104	0.0034	85	0.22745
53	182.7104	191.7104	0.0034	86	-0.11769
53	182.7104	191.7104	0.0034	81	0.26651
53	182.7104	191.7104	0.0034	82	0.28065
53	182.7104	191.7104	0.0034	85	0.3743
53	182.7104	191.7104	0.0034	86	-0.20096
53	182.7104	191.7104	0.0034	87	0.16141
54	182.7107	191.7107	0.0035	81	-0.26659
54	182.7107	191.7107	0.0035	82	0.28061
54	182.7107	191.7107	0.0035	85	0.37533
54	182.7107	191.7107	0.0035	86	0.20091
54	182.7107	191.7107	0.0035	87	-0.16183

54	182.7107	191.7107	0.0035	81	0.15519
54	182.7107	191.7107	0.0035	82	-0.16981
54	182.7107	191.7107	0.0035	85	-0.22715
54	182.7107	191.7107	0.0035	86	-0.11802
55	182.7184	191.7184	0.0057	81	0.14187
55	182.7184	191.7184	0.0057	82	0.10963
55	182.7184	191.7184	0.0057	85	0.15177
55	182.7184	191.7184	0.0057	86	-0.10749
55	182.7184	191.7184	0.0057	81	0.26949
55	182.7184	191.7184	0.0057	82	0.30257
55	182.7184	191.7184	0.0057	85	0.4188
55	182.7184	191.7184	0.0057	86	-0.20525
55	182.7184	191.7184	0.0057	87	0.16844
56	182.7188	191.7188	0.0043	81	-0.26896
56	182.7188	191.7188	0.0043	82	0.30225
56	182.7188	191.7188	0.0043	85	0.41963
56	182.7188	191.7188	0.0043	86	0.2053
56	182.7188	191.7188	0.0043	87	-0.16896
56	182.7188	191.7188	0.0043	81	0.14027
56	182.7188	191.7188	0.0043	82	-0.10923
56	182.7188	191.7188	0.0043	85	-0.15162
56	182.7188	191.7188	0.0043	86	-0.10763
57	182.7386	191.7386	0.0054	80	0.14372
57	182.7386	191.7386	0.0054	80	0.5835
57	182.7386	191.7386	0.0054	83	0.24377
57	182.7386	191.7386	0.0054	84	-0.22837
58	182.7393	191.7393	0.0053	80	0.58353
58	182.7393	191.7393	0.0053	83	-0.24434
58	182.7393	191.7393	0.0053	84	0.22876
58	182.7393	191.7393	0.0053	80	-0.14599
59	182.7406	191.7406	0.0088	80	0.60023
59	182.7406	191.7406	0.0088	83	0.25159
59	182.7406	191.7406	0.0088	84	-0.23535
60	182.7414	191.7414	0.0089	80	0.6008
60	182.7414	191.7414	0.0089	83	-0.25242
60	182.7414	191.7414	0.0089	84	0.23596
61	182.7593	191.7593	0.0006	81	-0.35396
61	182.7593	191.7593	0.0006	82	0.53468
61	182.7593	191.7593	0.0006	85	-0.19909
61	182.7593	191.7593	0.0006	87	-0.18942
62	182.7598	191.7598	0.0006	81	0.35536
62	182.7598	191.7598	0.0006	82	0.53473
62	182.7598	191.7598	0.0006	85	-0.19708
62	182.7598	191.7598	0.0006	87	0.18872

63	182.8047	191.8047	0.0078	82	-0.15343
63	182.8047	191.8047	0.0078	81	-0.26345
63	182.8047	191.8047	0.0078	82	0.5683
63	182.8047	191.8047	0.0078	85	-0.19132
63	182.8047	191.8047	0.0078	87	0.15959
64	182.8054	191.8054	0.0075	81	0.26304
64	182.8054	191.8054	0.0075	82	0.56826
64	182.8054	191.8054	0.0075	85	-0.19184
64	182.8054	191.8054	0.0075	87	-0.15929
64	182.8054	191.8054	0.0075	82	0.1534
65	182.8287	191.8287	0.002	81	-0.40256
65	182.8287	191.8287	0.002	85	0.49839
65	182.8287	191.8287	0.002	87	0.22694
65	182.8287	191.8287	0.002	89	0.14821
66	182.829	191.829	0.002	81	0.40188
66	182.829	191.829	0.002	85	0.49858
66	182.829	191.829	0.002	87	-0.22785
66	182.829	191.829	0.002	89	-0.14876
67	182.8379	191.8379	0.0021	80	0.10406
67	182.8379	191.8379	0.0021	80	0.10733
67	182.8379	191.8379	0.0021	83	0.30896
67	182.8379	191.8379	0.0021	80	0.178
67	182.8379	191.8379	0.0021	83	0.5241
67	182.8379	191.8379	0.0021	88	-0.12882
67	182.8379	191.8379	0.0021	90	0.10038
68	182.8385	191.8385	0.0025	80	0.14896
68	182.8385	191.8385	0.0025	80	-0.17766
68	182.8385	191.8385	0.0025	83	0.50984
68	182.8385	191.8385	0.0025	88	-0.12418
68	182.8385	191.8385	0.0025	90	-0.10134
68	182.8385	191.8385	0.0025	80	0.10151
68	182.8385	191.8385	0.0025	83	-0.31737
69	182.839	191.839	0.0001	80	0.10931
69	182.839	191.839	0.0001	83	0.2068
69	182.839	191.839	0.0001	84	-0.20806
69	182.839	191.839	0.0001	80	0.57567
69	182.839	191.839	0.0001	83	0.18068
70	182.8399	191.8399	0.0046	80	0.56955
70	182.8399	191.8399	0.0046	83	0.20625
70	182.8399	191.8399	0.0046	84	-0.20721
70	182.8399	191.8399	0.0046	83	-0.18579
71	182.8418	191.8418	0.0359	80	0.54965
71	182.8418	191.8418	0.0359	80	0.22243
71	182.8418	191.8418	0.0359	83	-0.1934

71	182.8418	191.8418	0.0359	84	0.19459
71	182.8418	191.8418	0.0359	83	-0.1697
72	182.8428	191.8428	0.0027	80	-0.17977
72	182.8428	191.8428	0.0027	83	-0.15123
72	182.8428	191.8428	0.0027	84	0.15213
72	182.8428	191.8428	0.0027	80	0.40565
72	182.8428	191.8428	0.0027	80	0.13743
72	182.8428	191.8428	0.0027	80	-0.12065
72	182.8428	191.8428	0.0027	83	0.16413
72	182.8428	191.8428	0.0027	80	0.12068
72	182.8428	191.8428	0.0027	83	0.34691
73	182.8428	191.8428	0.0034	80	0.13226
73	182.8428	191.8428	0.0034	83	0.12716
73	182.8428	191.8428	0.0034	84	-0.12795
73	182.8428	191.8428	0.0034	80	-0.38599
73	182.8428	191.8428	0.0034	80	-0.12684
73	182.8428	191.8428	0.0034	83	0.12485
73	182.8428	191.8428	0.0034	80	0.12866
73	182.8428	191.8428	0.0034	83	0.42477
73	182.8428	191.8428	0.0034	88	-0.10455
74	182.8434	191.8434	0.0069	80	0.15246
74	182.8434	191.8434	0.0069	80	-0.18018
74	182.8434	191.8434	0.0069	83	0.55582
74	182.8434	191.8434	0.0069	88	-0.1356
74	182.8434	191.8434	0.0069	90	-0.10364
74	182.8434	191.8434	0.0069	83	-0.21245
75	183.003	192.003	0.0081	81	-0.17948
75	183.003	192.003	0.0081	85	-0.15368
75	183.003	192.003	0.0081	80	-0.14611
75	183.003	192.003	0.0081	84	0.26379
75	183.003	192.003	0.0081	80	-0.21753
75	183.003	192.003	0.0081	84	0.50172
75	183.003	192.003	0.0081	88	-0.12983
76	183.0033	192.0033	0.0105	81	-0.21492
76	183.0033	192.0033	0.0105	85	0.17742
76	183.0033	192.0033	0.0105	80	0.21213
76	183.0033	192.0033	0.0105	84	0.48293
76	183.0033	192.0033	0.0105	88	-0.12413
76	183.0033	192.0033	0.0105	80	-0.13915
76	183.0033	192.0033	0.0105	84	-0.2608
77	183.0074	192.0074	0.0078	85	0.19794
77	183.0074	192.0074	0.0078	81	0.45278
77	183.0074	192.0074	0.0078	85	-0.32043
77	183.0074	192.0074	0.0078	86	-0.11378

77	183.0074	192.0074	0.0078	87	0.1835
77	183.0074	192.0074	0.0078	84	0.26211
78	183.0078	192.0078	0.0025	81	0.46695
78	183.0078	192.0078	0.0025	85	0.33297
78	183.0078	192.0078	0.0025	86	-0.11799
78	183.0078	192.0078	0.0025	87	0.18964
78	183.0078	192.0078	0.0025	85	0.20379
78	183.0078	192.0078	0.0025	84	0.22218
79	183.0084	192.0084	0.0004	80	-0.13112
79	183.0084	192.0084	0.0004	84	0.20693
79	183.0084	192.0084	0.0004	80	-0.24983
79	183.0084	192.0084	0.0004	84	0.57302
79	183.0084	192.0084	0.0004	88	-0.14809
80	183.0089	192.0089	0.0005	80	0.24988
80	183.0089	192.0089	0.0005	84	0.57404
80	183.0089	192.0089	0.0005	88	-0.14726
80	183.0089	192.0089	0.0005	80	-0.13115
80	183.0089	192.0089	0.0005	84	-0.20726
81	183.0163	192.0163	0.0017	81	0.4202
81	183.0163	192.0163	0.0017	82	0.40514
81	183.0163	192.0163	0.0017	85	0.27208
81	183.0163	192.0163	0.0017	86	-0.21625
81	183.0163	192.0163	0.0017	87	0.1435
81	183.0163	192.0163	0.0017	91	0.10362
82	183.0166	192.0166	0.0018	81	0.41963
82	183.0166	192.0166	0.0018	82	-0.40498
82	183.0166	192.0166	0.0018	85	-0.27274
82	183.0166	192.0166	0.0018	86	-0.21659
82	183.0166	192.0166	0.0018	87	0.14407
82	183.0166	192.0166	0.0018	91	-0.10335
83	183.022	192.022	0.0078	80	0.22003
83	183.022	192.022	0.0078	83	0.57028
83	183.022	192.022	0.0078	84	0.17939
83	183.022	192.022	0.0078	88	-0.2265
83	183.022	192.022	0.0078	90	0.18532
84	183.023	192.023	0.0083	80	-0.21972
84	183.023	192.023	0.0083	83	0.57083
84	183.023	192.023	0.0083	84	0.17949
84	183.023	192.023	0.0083	88	-0.22449
84	183.023	192.023	0.0083	90	-0.18701
85	183.0894	192.0894	0.0038	85	-0.11167
85	183.0894	192.0894	0.0038	81	0.3933
85	183.0894	192.0894	0.0038	82	0.3353
85	183.0894	192.0894	0.0038	85	0.39841

85	183.0894	192.0894	0.0038	86	0.1234
85	183.0894	192.0894	0.0038	87	-0.12219
86	183.0902	192.0902	0.0035	81	-0.39432
86	183.0902	192.0902	0.0035	82	0.33559
86	183.0902	192.0902	0.0035	85	0.39793
86	183.0902	192.0902	0.0035	86	-0.12317
86	183.0902	192.0902	0.0035	87	0.12166
86	183.0902	192.0902	0.0035	85	0.11127
87	183.1297	192.1297	0.0062	80	0.14869
87	183.1297	192.1297	0.0062	80	-0.20428
87	183.1297	192.1297	0.0062	83	0.63777
87	183.1297	192.1297	0.0062	88	-0.11264
88	183.13	192.13	0.0012	80	0.20484
88	183.13	192.13	0.0012	83	0.63753
88	183.13	192.13	0.0012	88	-0.11331
88	183.13	192.13	0.0012	80	0.14849
89	183.1944	192.1944	0.0013	80	0.49855
89	183.1944	192.1944	0.0013	83	0.23499
89	183.1944	192.1944	0.0013	84	-0.1803
89	183.1944	192.1944	0.0013	80	0.31402
89	183.1944	192.1944	0.0013	83	-0.17669
89	183.1944	192.1944	0.0013	84	0.13546
90	183.1945	192.1945	0.0371	80	-0.31421
90	183.1945	192.1945	0.0371	83	-0.17634
90	183.1945	192.1945	0.0371	84	0.1353
90	183.1945	192.1945	0.0371	80	0.49823
90	183.1945	192.1945	0.0371	83	-0.23542
90	183.1945	192.1945	0.0371	84	0.18048
91	183.2245	192.2245	0.0271	81	0.26513
91	183.2245	192.2245	0.0271	82	-0.26688
91	183.2245	192.2245	0.0271	85	0.13468
91	183.2245	192.2245	0.0271	87	0.17701
91	183.2245	192.2245	0.0271	81	0.14891
91	183.2245	192.2245	0.0271	82	0.42701
91	183.2245	192.2245	0.0271	85	-0.2135
92	183.2247	192.2247	0.0001	81	-0.1431
92	183.2247	192.2247	0.0001	82	0.42023
92	183.2247	192.2247	0.0001	85	-0.21053
92	183.2247	192.2247	0.0001	81	0.26199
92	183.2247	192.2247	0.0001	82	0.26767
92	183.2247	192.2247	0.0001	85	-0.13287
92	183.2247	192.2247	0.0001	87	0.17356
93	183.2251	192.2251	0.0038	81	0.14153
93	183.2251	192.2251	0.0038	82	-0.19061

93	183.2251	192.2251	0.0038	81	0.26675
93	183.2251	192.2251	0.0038	82	0.46539
93	183.2251	192.2251	0.0038	85	-0.23538
93	183.2251	192.2251	0.0038	87	0.17968
93	183.2251	192.2251	0.0038	83	-0.11257
94	183.2253	192.2253	0.0008	81	-0.26146
94	183.2253	192.2253	0.0008	82	0.45915
94	183.2253	192.2253	0.0008	85	-0.23373
94	183.2253	192.2253	0.0008	87	-0.17715
94	183.2253	192.2253	0.0008	81	0.13731
94	183.2253	192.2253	0.0008	82	0.18811
94	183.2253	192.2253	0.0008	82	0.10389
95	183.2526	192.2526	0.0023	81	0.10399
95	183.2526	192.2526	0.0023	85	-0.1696
95	183.2526	192.2526	0.0023	86	0.38092
95	183.2526	192.2526	0.0023	89	0.25714
95	183.2526	192.2526	0.0023	91	0.26034
95	183.2526	192.2526	0.0023	86	-0.2848
95	183.2526	192.2526	0.0023	89	-0.19181
95	183.2526	192.2526	0.0023	91	-0.12039
96	183.2531	192.2531	0.0032	86	0.2839
96	183.2531	192.2531	0.0032	89	0.18966
96	183.2531	192.2531	0.0032	91	-0.12101
96	183.2531	192.2531	0.0032	81	0.10322
96	183.2531	192.2531	0.0032	85	0.16883
96	183.2531	192.2531	0.0032	86	0.37988
96	183.2531	192.2531	0.0032	89	0.25319
96	183.2531	192.2531	0.0032	91	-0.26093
97	183.2547	192.2547	0.0026	80	-0.28422
97	183.2547	192.2547	0.0026	83	-0.17754
97	183.2547	192.2547	0.0026	84	0.58301
97	183.2547	192.2547	0.0026	88	-0.16127
97	183.2547	192.2547	0.0026	90	0.10491
98	183.2553	192.2553	0.0025	80	0.28509
98	183.2553	192.2553	0.0025	83	-0.17852
98	183.2553	192.2553	0.0025	84	0.58592
98	183.2553	192.2553	0.0025	88	-0.16081
98	183.2553	192.2553	0.0025	90	-0.10655
99	183.26	192.26	0.0033	81	0.11558
99	183.26	192.26	0.0033	85	-0.17257
99	183.26	192.26	0.0033	86	0.42949
99	183.26	192.26	0.0033	87	-0.10759
99	183.26	192.26	0.0033	89	0.28703
99	183.26	192.26	0.0033	91	0.26736

99	183.26	192.26	0.0033	86	-0.21281
99	183.26	192.26	0.0033	89	-0.14204
99	183.26	192.26	0.0033	91	-0.1036
100	183.2605	192.2605	0.004	86	0.21308
100	183.2605	192.2605	0.004	89	0.14089
100	183.2605	192.2605	0.004	91	-0.10448
100	183.2605	192.2605	0.004	81	0.11526
100	183.2605	192.2605	0.004	85	0.17262
100	183.2605	192.2605	0.004	86	0.43026
100	183.2605	192.2605	0.004	87	-0.10756
100	183.2605	192.2605	0.004	89	0.28412
100	183.2605	192.2605	0.004	91	-0.26927
101	183.3088	192.3088	0.0019	82	-0.17344
101	183.3088	192.3088	0.0019	80	0.1345
101	183.3088	192.3088	0.0019	84	0.62729
101	183.3088	192.3088	0.0019	88	-0.16009
102	183.3092	192.3092	0.0007	82	-0.23489
102	183.3092	192.3092	0.0007	80	-0.13048
102	183.3092	192.3092	0.0007	84	0.60457
102	183.3092	192.3092	0.0007	88	-0.15516
103	183.311	192.311	0.0005	83	0.12184
103	183.311	192.311	0.0005	83	0.11583
103	183.311	192.311	0.0005	82	-0.12596
103	183.311	192.311	0.0005	82	0.6305
104	183.3115	192.3115	0.0028	83	0.11672
104	183.3115	192.3115	0.0028	83	-0.10805
104	183.3115	192.3115	0.0028	82	0.62312
104	183.3115	192.3115	0.0028	82	0.12937
105	183.3121	192.3121	0.0004	82	0.59413
105	183.3121	192.3121	0.0004	82	-0.10222
105	183.3121	192.3121	0.0004	83	-0.13001
105	183.3121	192.3121	0.0004	84	0.22003
106	183.3125	192.3125	0.0019	82	0.61491
106	183.3125	192.3125	0.0019	83	0.14879
106	183.3125	192.3125	0.0019	84	0.10284
106	183.3125	192.3125	0.0019	84	0.16519
107	183.3182	192.3182	0.0231	81	-0.14927
107	183.3182	192.3182	0.0231	85	-0.20228
107	183.3182	192.3182	0.0231	81	0.18041
107	183.3182	192.3182	0.0231	87	-0.1089
107	183.3182	192.3182	0.0231	81	0.29303
107	183.3182	192.3182	0.0231	82	0.10528
107	183.3182	192.3182	0.0231	85	0.44073
107	183.3182	192.3182	0.0231	87	-0.17745

108	183.3184	192.3184	0.0071	81	0.14384
108	183.3184	192.3184	0.0071	85	-0.18375
108	183.3184	192.3184	0.0071	81	-0.29411
108	183.3184	192.3184	0.0071	82	0.10911
108	183.3184	192.3184	0.0071	85	0.45103
108	183.3184	192.3184	0.0071	87	0.1771
108	183.3184	192.3184	0.0071	81	0.18764
108	183.3184	192.3184	0.0071	87	-0.11339
109	183.3187	192.3187	0.0164	81	0.33393
109	183.3187	192.3187	0.0164	82	0.10708
109	183.3187	192.3187	0.0164	85	0.43546
109	183.3187	192.3187	0.0164	87	-0.20088
109	183.3187	192.3187	0.0164	89	-0.11121
109	183.3187	192.3187	0.0164	81	0.13345
109	183.3187	192.3187	0.0164	85	0.20966
110	183.3189	192.3189	0.0082	81	-0.33637
110	183.3189	192.3189	0.0082	82	0.11061
110	183.3189	192.3189	0.0082	85	0.44241
110	183.3189	192.3189	0.0082	87	0.20132
110	183.3189	192.3189	0.0082	89	0.11167
110	183.3189	192.3189	0.0082	81	-0.13304
110	183.3189	192.3189	0.0082	85	0.18641
111	183.4078	192.4078	0.0102	81	0.15413
111	183.4078	192.4078	0.0102	82	-0.15774
111	183.4078	192.4078	0.0102	85	-0.1411
111	183.4078	192.4078	0.0102	86	0.43209
111	183.4078	192.4078	0.0102	87	-0.13476
111	183.4078	192.4078	0.0102	89	0.35013
111	183.4078	192.4078	0.0102	91	0.29322
112	183.4081	192.4081	0.0145	81	0.15369
112	183.4081	192.4081	0.0145	82	0.15764
112	183.4081	192.4081	0.0145	85	0.14098
112	183.4081	192.4081	0.0145	86	0.43268
112	183.4081	192.4081	0.0145	87	-0.13497
112	183.4081	192.4081	0.0145	89	0.34731
112	183.4081	192.4081	0.0145	91	-0.2959
113	183.433	192.433	0.0256	81	0.11842
113	183.433	192.433	0.0256	85	0.18316
113	183.433	192.433	0.0256	91	-0.11205
113	183.433	192.433	0.0256	81	0.62378
113	183.433	192.433	0.0256	89	-0.13616
114	183.4337	192.4337	0	81	-0.1249
114	183.4337	192.4337	0	81	0.61275
114	183.4337	192.4337	0	89	-0.13388

114	183.4337	192.4337	0	81	-0.11574
114	183.4337	192.4337	0	85	0.18355
114	183.4337	192.4337	0	91	-0.11344
115	183.4339	192.4339	0	81	0.62769
115	183.4339	192.4339	0	85	0.11956
115	183.4339	192.4339	0	89	-0.13864
115	183.4339	192.4339	0	85	-0.14556
116	183.434	192.434	0.0001	85	-0.14499
116	183.434	192.434	0.0001	81	0.61801
116	183.434	192.434	0.0001	85	-0.11884
116	183.434	192.434	0.0001	89	-0.13539
116	183.434	192.434	0.0001	81	0.12485
117	183.4475	192.4475	0.0033	82	-0.10687
117	183.4475	192.4475	0.0033	86	-0.17686
117	183.4475	192.4475	0.0033	87	-0.1892
117	183.4475	192.4475	0.0033	89	0.1415
117	183.4475	192.4475	0.0033	91	-0.1392
117	183.4475	192.4475	0.0033	88	0.20738
117	183.4475	192.4475	0.0033	90	-0.15118
117	183.4475	192.4475	0.0033	83	0.10696
117	183.4475	192.4475	0.0033	84	0.16157
117	183.4475	192.4475	0.0033	88	0.40043
117	183.4475	192.4475	0.0033	90	-0.2217
118	183.4483	192.4483	0.0043	82	0.11229
118	183.4483	192.4483	0.0043	86	-0.1866
118	183.4483	192.4483	0.0043	87	-0.19713
118	183.4483	192.4483	0.0043	89	0.1489
118	183.4483	192.4483	0.0043	91	0.14547
118	183.4483	192.4483	0.0043	83	0.10756
118	183.4483	192.4483	0.0043	84	0.16282
118	183.4483	192.4483	0.0043	88	0.40294
118	183.4483	192.4483	0.0043	90	0.22948
118	183.4483	192.4483	0.0043	88	-0.21365
118	183.4483	192.4483	0.0043	90	-0.15284
119	183.4487	192.4487	0.0003	88	0.14779
119	183.4487	192.4487	0.0003	90	-0.11063
119	183.4487	192.4487	0.0003	83	0.10111
119	183.4487	192.4487	0.0003	84	0.15715
119	183.4487	192.4487	0.0003	88	0.38989
119	183.4487	192.4487	0.0003	90	-0.2188
119	183.4487	192.4487	0.0003	86	-0.10597
119	183.4487	192.4487	0.0003	87	-0.10742
119	183.4487	192.4487	0.0003	82	-0.10933
119	183.4487	192.4487	0.0003	86	-0.20259

119	183.4487	192.4487	0.0003	87	-0.2051
119	183.4487	192.4487	0.0003	89	0.15488
119	183.4487	192.4487	0.0003	91	-0.14836
120	183.4492	192.4492	0.0001	83	0.10196
120	183.4492	192.4492	0.0001	84	0.15904
120	183.4492	192.4492	0.0001	88	0.39435
120	183.4492	192.4492	0.0001	90	0.22424
120	183.4492	192.4492	0.0001	88	-0.14774
120	183.4492	192.4492	0.0001	90	-0.1141
120	183.4492	192.4492	0.0001	82	0.11433
120	183.4492	192.4492	0.0001	86	-0.21429
120	183.4492	192.4492	0.0001	87	-0.21447
120	183.4492	192.4492	0.0001	89	0.16297
120	183.4492	192.4492	0.0001	91	0.15385
120	183.4492	192.4492	0.0001	86	0.11266
120	183.4492	192.4492	0.0001	87	0.11263
120	183.4492	192.4492	0.0001	91	-0.10361
121	183.4947	192.4947	0.0038	90	-0.1168
121	183.4947	192.4947	0.0038	83	0.11407
121	183.4947	192.4947	0.0038	84	0.13974
121	183.4947	192.4947	0.0038	88	0.31931
121	183.4947	192.4947	0.0038	90	-0.19271
121	183.4947	192.4947	0.0038	86	0.14575
121	183.4947	192.4947	0.0038	87	0.1054
121	183.4947	192.4947	0.0038	91	0.11979
121	183.4947	192.4947	0.0038	86	0.33316
121	183.4947	192.4947	0.0038	87	0.24041
121	183.4947	192.4947	0.0038	89	-0.18794
121	183.4947	192.4947	0.0038	91	0.15395
122	183.4951	192.4951	0.0063	83	0.11503
122	183.4951	192.4951	0.0063	84	0.14139
122	183.4951	192.4951	0.0063	88	0.32262
122	183.4951	192.4951	0.0063	90	0.19833
122	183.4951	192.4951	0.0063	90	-0.11842
122	183.4951	192.4951	0.0063	86	0.33093
122	183.4951	192.4951	0.0063	87	0.23753
122	183.4951	192.4951	0.0063	89	-0.18713
122	183.4951	192.4951	0.0063	91	-0.14988
122	183.4951	192.4951	0.0063	86	-0.14385
122	183.4951	192.4951	0.0063	87	-0.10303
122	183.4951	192.4951	0.0063	91	0.11815
123	183.4984	192.4984	0.0008	86	0.11108
123	183.4984	192.4984	0.0008	91	0.1102
123	183.4984	192.4984	0.0008	82	0.10084

123	183.4984	192.4984	0.0008	86	0.36892
123	183.4984	192.4984	0.0008	87	0.27735
123	183.4984	192.4984	0.0008	89	-0.21538
123	183.4984	192.4984	0.0008	91	0.18563
123	183.4984	192.4984	0.0008	88	0.11397
123	183.4984	192.4984	0.0008	90	-0.12115
123	183.4984	192.4984	0.0008	84	0.11354
123	183.4984	192.4984	0.0008	88	0.25979
123	183.4984	192.4984	0.0008	90	-0.15497
124	183.4986	192.4986	0.0015	86	0.36724
124	183.4986	192.4986	0.0015	87	0.27465
124	183.4986	192.4986	0.0015	89	-0.21515
124	183.4986	192.4986	0.0015	91	-0.18182
124	183.4986	192.4986	0.0015	86	-0.11014
124	183.4986	192.4986	0.0015	91	0.10902
124	183.4986	192.4986	0.0015	84	0.11509
124	183.4986	192.4986	0.0015	88	0.26297
124	183.4986	192.4986	0.0015	90	0.15974
124	183.4986	192.4986	0.0015	88	-0.11623
124	183.4986	192.4986	0.0015	90	-0.12349
125	183.5207	192.5207	0.0072	81	-0.12473
125	183.5207	192.5207	0.0072	83	0.15337
125	183.5207	192.5207	0.0072	81	0.21241
125	183.5207	192.5207	0.0072	81	0.37147
125	183.5207	192.5207	0.0072	82	-0.30092
125	183.5207	192.5207	0.0072	83	-0.16248
125	183.5207	192.5207	0.0072	85	-0.18643
125	183.5207	192.5207	0.0072	86	-0.14008
125	183.5207	192.5207	0.0072	87	0.10112
125	183.5207	192.5207	0.0072	91	-0.10935
126	183.5209	192.5209	0.0024	81	-0.13731
126	183.5209	192.5209	0.0024	83	-0.14138
126	183.5209	192.5209	0.0024	81	0.36533
126	183.5209	192.5209	0.0024	82	0.30004
126	183.5209	192.5209	0.0024	83	0.17354
126	183.5209	192.5209	0.0024	85	0.1863
126	183.5209	192.5209	0.0024	86	-0.1374
126	183.5209	192.5209	0.0024	91	0.10957
126	183.5209	192.5209	0.0024	81	-0.21526
127	183.5213	192.5213	0.0032	81	0.10172
127	183.5213	192.5213	0.0032	81	0.41209
127	183.5213	192.5213	0.0032	82	-0.29409
127	183.5213	192.5213	0.0032	83	0.15701
127	183.5213	192.5213	0.0032	85	-0.18335

127	183.5213	192.5213	0.0032	86	-0.15519
127	183.5213	192.5213	0.0032	87	0.11215
127	183.5213	192.5213	0.0032	91	-0.10759
127	183.5213	192.5213	0.0032	81	0.11721
127	183.5213	192.5213	0.0032	83	0.15887
128	183.5216	192.5216	0.0023	81	0.40563
128	183.5216	192.5216	0.0023	82	0.29095
128	183.5216	192.5216	0.0023	83	-0.16938
128	183.5216	192.5216	0.0023	85	0.18139
128	183.5216	192.5216	0.0023	86	-0.15234
128	183.5216	192.5216	0.0023	87	0.11004
128	183.5216	192.5216	0.0023	91	0.10688
128	183.5216	192.5216	0.0023	81	-0.10538
128	183.5216	192.5216	0.0023	81	0.13144
128	183.5216	192.5216	0.0023	83	-0.14728
129	183.5335	192.5335	0.011	80	-0.13278
129	183.5335	192.5335	0.011	80	-0.13545
129	183.5335	192.5335	0.011	83	-0.21789
129	183.5335	192.5335	0.011	85	0.42697
129	183.5335	192.5335	0.011	81	-0.22057
129	183.5335	192.5335	0.011	85	-0.28155
129	183.5335	192.5335	0.011	86	0.11068
129	183.5335	192.5335	0.011	87	-0.19319
130	183.534	192.534	0.0035	81	0.13932
130	183.534	192.534	0.0035	87	0.11783
130	183.534	192.534	0.0035	81	0.18097
130	183.534	192.534	0.0035	85	0.5012
130	183.534	192.534	0.0035	87	0.15435
130	183.534	192.534	0.0035	83	-0.16423
130	183.534	192.534	0.0035	80	0.17801
130	183.534	192.534	0.0035	83	0.14429
130	183.534	192.534	0.0035	81	-0.1114
131	183.5344	192.5344	0.002	80	-0.13938
131	183.5344	192.5344	0.002	83	0.22787
131	183.5344	192.5344	0.002	80	0.13574
131	183.5344	192.5344	0.002	81	-0.21585
131	183.5344	192.5344	0.002	85	0.2798
131	183.5344	192.5344	0.002	86	0.10901
131	183.5344	192.5344	0.002	87	-0.19092
131	183.5344	192.5344	0.002	85	0.43041
132	183.5347	192.5347	0.0095	81	-0.18437
132	183.5347	192.5347	0.0095	85	0.50113
132	183.5347	192.5347	0.0095	87	-0.15777
132	183.5347	192.5347	0.0095	81	0.13327

132	183.5347	192.5347	0.0095	87	0.11496
132	183.5347	192.5347	0.0095	80	-0.18214
132	183.5347	192.5347	0.0095	83	0.14826
132	183.5347	192.5347	0.0095	83	0.17578
132	183.5347	192.5347	0.0095	81	0.10867
133	183.5979	192.5979	0.0004	81	0.10743
133	183.5979	192.5979	0.0004	82	0.20768
133	183.5979	192.5979	0.0004	80	0.20742
133	183.5979	192.5979	0.0004	82	-0.15105
133	183.5979	192.5979	0.0004	83	0.51543
133	183.5979	192.5979	0.0004	88	-0.15062
133	183.5979	192.5979	0.0004	90	0.15465
133	183.5979	192.5979	0.0004	83	-0.18543
134	183.5983	192.5983	0.0005	81	-0.108
134	183.5983	192.5983	0.0005	82	0.20995
134	183.5983	192.5983	0.0005	83	0.18589
134	183.5983	192.5983	0.0005	80	-0.20605
134	183.5983	192.5983	0.0005	82	-0.14406
134	183.5983	192.5983	0.0005	83	0.51453
134	183.5983	192.5983	0.0005	88	-0.14832
134	183.5983	192.5983	0.0005	90	-0.15472
135	183.5989	192.5989	0.0007	80	0.20983
135	183.5989	192.5989	0.0007	82	0.15859
135	183.5989	192.5989	0.0007	83	0.54704
135	183.5989	192.5989	0.0007	88	-0.15963
135	183.5989	192.5989	0.0007	90	0.15656
135	183.5989	192.5989	0.0007	81	0.1007
135	183.5989	192.5989	0.0007	82	0.1949
136	183.5993	192.5993	0.0006	80	-0.20876
136	183.5993	192.5993	0.0006	82	0.1538
136	183.5993	192.5993	0.0006	83	0.54607
136	183.5993	192.5993	0.0006	88	-0.15725
136	183.5993	192.5993	0.0006	90	-0.15687
136	183.5993	192.5993	0.0006	81	-0.10149
136	183.5993	192.5993	0.0006	82	0.19938
137	183.621	192.621	0.0001	85	-0.13473
137	183.621	192.621	0.0001	82	-0.11374
137	183.621	192.621	0.0001	85	-0.1973
137	183.621	192.621	0.0001	80	-0.14279
137	183.621	192.621	0.0001	83	-0.2276
137	183.621	192.621	0.0001	80	0.13587
137	183.621	192.621	0.0001	83	0.49401
137	183.621	192.621	0.0001	84	-0.1115
138	183.6218	192.6218	0.0002	82	-0.11702

138	183.6218	192.6218	0.0002	85	-0.20327
138	183.6218	192.6218	0.0002	83	0.21104
138	183.6218	192.6218	0.0002	80	-0.13229
138	183.6218	192.6218	0.0002	83	0.45193
138	183.6218	192.6218	0.0002	84	-0.10264
138	183.6218	192.6218	0.0002	85	0.13874
138	183.6218	192.6218	0.0002	80	-0.1159
138	183.6218	192.6218	0.0002	83	0.24274
139	183.622	192.622	0.0003	83	0.20885
139	183.622	192.622	0.0003	80	0.16956
139	183.622	192.622	0.0003	83	0.47052
139	183.622	192.622	0.0003	84	-0.10558
139	183.622	192.622	0.0003	85	-0.14376
139	183.622	192.622	0.0003	83	-0.21051
139	183.622	192.622	0.0003	82	-0.10988
139	183.622	192.622	0.0003	85	-0.18952
140	183.6227	192.6227	0.0004	80	-0.17897
140	183.6227	192.6227	0.0004	83	0.51193
140	183.6227	192.6227	0.0004	84	-0.11542
140	183.6227	192.6227	0.0004	83	-0.18772
140	183.6227	192.6227	0.0004	82	-0.10754
140	183.6227	192.6227	0.0004	85	-0.18508
140	183.6227	192.6227	0.0004	85	-0.14774
141	183.6231	192.6231	0.0063	83	0.23927
141	183.6231	192.6231	0.0063	84	0.26865
141	183.6231	192.6231	0.0063	88	0.50654
141	183.6231	192.6231	0.0063	90	-0.32192
142	183.6242	192.6242	0.0068	83	0.23899
142	183.6242	192.6242	0.0068	84	0.26817
142	183.6242	192.6242	0.0068	88	0.50449
142	183.6242	192.6242	0.0068	90	0.32621
143	183.664	192.664	0.001	85	-0.19803
143	183.664	192.664	0.001	86	0.49246
143	183.664	192.664	0.001	87	-0.21465
143	183.664	192.664	0.001	89	0.27276
143	183.664	192.664	0.001	91	0.25657
144	183.6653	192.6653	0.0007	85	0.19832
144	183.6653	192.6653	0.0007	86	0.4932
144	183.6653	192.6653	0.0007	87	-0.21471
144	183.6653	192.6653	0.0007	89	0.26975
144	183.6653	192.6653	0.0007	91	-0.25798
145	183.6899	192.6899	0.0001	85	0.26573
145	183.6899	192.6899	0.0001	87	0.40543
145	183.6899	192.6899	0.0001	89	0.28724

145	183.6899	192.6899	0.0001	91	0.15274
145	183.6899	192.6899	0.0001	85	-0.13515
145	183.6899	192.6899	0.0001	87	-0.28036
145	183.6899	192.6899	0.0001	89	-0.19864
146	183.6905	192.6905	0.0001	85	-0.13494
146	183.6905	192.6905	0.0001	87	0.28029
146	183.6905	192.6905	0.0001	89	0.19829
146	183.6905	192.6905	0.0001	85	-0.26531
146	183.6905	192.6905	0.0001	87	0.40518
146	183.6905	192.6905	0.0001	89	0.28665
146	183.6905	192.6905	0.0001	91	-0.15525
147	183.6958	192.6958	0.0004	85	0.27685
147	183.6958	192.6958	0.0004	86	-0.10501
147	183.6958	192.6958	0.0004	87	0.4428
147	183.6958	192.6958	0.0004	89	0.31447
147	183.6958	192.6958	0.0004	91	0.15955
147	183.6958	192.6958	0.0004	85	-0.10991
147	183.6958	192.6958	0.0004	87	-0.21491
147	183.6958	192.6958	0.0004	89	-0.15265
148	183.6964	192.6964	0.0005	85	-0.10972
148	183.6964	192.6964	0.0005	87	0.21487
148	183.6964	192.6964	0.0005	89	0.15237
148	183.6964	192.6964	0.0005	85	-0.27641
148	183.6964	192.6964	0.0005	86	-0.1049
148	183.6964	192.6964	0.0005	87	0.44251
148	183.6964	192.6964	0.0005	89	0.31385
148	183.6964	192.6964	0.0005	91	-0.16228
149	183.7097	192.7097	0.0035	82	0.14164
149	183.7097	192.7097	0.0035	86	0.46813
149	183.7097	192.7097	0.0035	87	0.33844
149	183.7097	192.7097	0.0035	89	-0.25797
149	183.7097	192.7097	0.0035	91	0.24808
150	183.7098	192.7098	0.0043	82	-0.14174
150	183.7098	192.7098	0.0043	86	0.4686
150	183.7098	192.7098	0.0043	87	0.33798
150	183.7098	192.7098	0.0043	89	-0.25987
150	183.7098	192.7098	0.0043	91	-0.24583
151	183.7656	192.7656	0.0009	82	-0.10191
151	183.7656	192.7656	0.0009	80	0.13124
151	183.7656	192.7656	0.0009	84	-0.13987
151	183.7656	192.7656	0.0009	83	0.15793
151	183.7656	192.7656	0.0009	84	0.5902
151	183.7656	192.7656	0.0009	88	-0.15526
152	183.7664	192.7664	0.0004	82	-0.10433

152	183.7664	192.7664	0.0004	83	0.15758
152	183.7664	192.7664	0.0004	84	0.58666
152	183.7664	192.7664	0.0004	88	-0.15376
152	183.7664	192.7664	0.0004	80	0.1284
152	183.7664	192.7664	0.0004	84	0.13999
153	183.7666	192.7666	0.0023	84	0.15191
153	183.7666	192.7666	0.0023	80	-0.13314
153	183.7666	192.7666	0.0023	83	0.15598
153	183.7666	192.7666	0.0023	84	0.57842
153	183.7666	192.7666	0.0023	88	-0.15245
153	183.7666	192.7666	0.0023	82	-0.10395
153	183.7666	192.7666	0.0023	84	0.11027
154	183.7672	192.7672	0.0015	80	0.13562
154	183.7672	192.7672	0.0015	83	0.15691
154	183.7672	192.7672	0.0015	84	0.58061
154	183.7672	192.7672	0.0015	88	-0.15197
154	183.7672	192.7672	0.0015	84	-0.15323
154	183.7672	192.7672	0.0015	82	-0.10528
154	183.7672	192.7672	0.0015	84	0.11882
155	183.7811	192.7811	0.0007	80	-0.27652
155	183.7811	192.7811	0.0007	84	0.44909
155	183.7811	192.7811	0.0007	88	-0.11864
155	183.7811	192.7811	0.0007	90	0.10694
155	183.7811	192.7811	0.0007	84	-0.36334
156	183.7813	192.7813	0.0024	84	0.36303
156	183.7813	192.7813	0.0024	80	0.27626
156	183.7813	192.7813	0.0024	84	0.44917
156	183.7813	192.7813	0.0024	88	-0.11727
156	183.7813	192.7813	0.0024	90	-0.1078
157	183.7827	192.7827	0.0057	81	-0.15895
157	183.7827	192.7827	0.0057	82	0.15104
157	183.7827	192.7827	0.0057	84	-0.10794
157	183.7827	192.7827	0.0057	80	-0.26461
157	183.7827	192.7827	0.0057	84	0.53695
157	183.7827	192.7827	0.0057	88	-0.14203
157	183.7827	192.7827	0.0057	90	0.10231
158	183.7828	192.7828	0.0034	81	0.10661
158	183.7828	192.7828	0.0034	82	0.10298
158	183.7828	192.7828	0.0034	84	-0.1181
158	183.7828	192.7828	0.0034	80	0.27148
158	183.7828	192.7828	0.0034	83	-0.10164
158	183.7828	192.7828	0.0034	84	0.55265
158	183.7828	192.7828	0.0034	88	-0.14478
158	183.7828	192.7828	0.0034	90	-0.10605

159	183.7839	192.7839	0.0009	81	0.48185
159	183.7839	192.7839	0.0009	82	-0.44935
159	183.7839	192.7839	0.0009	84	0.18258
160	183.7845	192.7845	0.0013	81	0.49696
160	183.7845	192.7845	0.0013	82	0.46283
160	183.7845	192.7845	0.0013	84	-0.12249
161	183.8271	192.8271	0.0058	83	0.14819
161	183.8271	192.8271	0.0058	84	0.17234
161	183.8271	192.8271	0.0058	88	0.51445
161	183.8271	192.8271	0.0058	90	-0.35961
161	183.8271	192.8271	0.0058	88	0.18755
162	183.8275	192.8275	0.004	88	-0.18672
162	183.8275	192.8275	0.004	83	0.14774
162	183.8275	192.8275	0.004	84	0.17199
162	183.8275	192.8275	0.004	88	0.51201
162	183.8275	192.8275	0.004	90	0.36364
163	183.8409	192.8409	0.003	91	0.1029
163	183.8409	192.8409	0.003	82	-0.14208
163	183.8409	192.8409	0.003	86	0.44714
163	183.8409	192.8409	0.003	87	0.40067
163	183.8409	192.8409	0.003	89	-0.19922
163	183.8409	192.8409	0.003	91	-0.2269
164	183.8419	192.8419	0.0015	82	0.14195
164	183.8419	192.8419	0.0015	86	0.4464
164	183.8419	192.8419	0.0015	87	0.40129
164	183.8419	192.8419	0.0015	89	-0.19735
164	183.8419	192.8419	0.0015	91	0.22877
164	183.8419	192.8419	0.0015	91	0.10338
165	183.8904	192.8904	0.0242	81	-0.17024
165	183.8904	192.8904	0.0242	82	-0.21489
165	183.8904	192.8904	0.0242	85	0.53556
165	183.8904	192.8904	0.0242	87	0.30854
165	183.8904	192.8904	0.0242	89	0.13426
166	183.8912	192.8912	0.0186	81	0.16993
166	183.8912	192.8912	0.0186	82	-0.21585
166	183.8912	192.8912	0.0186	85	0.53493
166	183.8912	192.8912	0.0186	87	-0.30892
166	183.8912	192.8912	0.0186	89	-0.13472
167	183.9094	192.9094	0.0009	85	0.33749
167	183.9094	192.9094	0.0009	86	-0.12611
167	183.9094	192.9094	0.0009	87	0.49024
167	183.9094	192.9094	0.0009	89	0.31874
167	183.9094	192.9094	0.0009	91	0.14884
168	183.9097	192.9097	0.001	85	-0.33715

168	183.9097	192.9097	0.001	86	-0.12612
168	183.9097	192.9097	0.001	87	0.49017
168	183.9097	192.9097	0.001	89	0.31817
168	183.9097	192.9097	0.001	91	-0.15148
169	183.9805	192.9805	0.0001	88	-0.32555
169	183.9805	192.9805	0.0001	90	0.49926
169	183.9805	192.9805	0.0001	88	0.18581
169	183.9805	192.9805	0.0001	90	-0.31098
170	183.982	192.982	0.0002	88	0.18336
170	183.982	192.982	0.0002	90	0.31113
170	183.982	192.982	0.0002	88	0.32053
170	183.982	192.982	0.0002	90	0.5008
171	183.9853	192.9853	0.0028	81	0.12434
171	183.9853	192.9853	0.0028	86	0.26364
171	183.9853	192.9853	0.0028	87	-0.12911
171	183.9853	192.9853	0.0028	89	0.22182
171	183.9853	192.9853	0.0028	91	0.28927
171	183.9853	192.9853	0.0028	86	-0.21115
171	183.9853	192.9853	0.0028	87	0.10346
171	183.9853	192.9853	0.0028	89	-0.17674
171	183.9853	192.9853	0.0028	86	0.14666
171	183.9853	192.9853	0.0028	89	0.12303
171	183.9853	192.9853	0.0028	91	0.12685
171	183.9853	192.9853	0.0028	88	0.10809
171	183.9853	192.9853	0.0028	90	-0.15434
172	183.9855	192.9855	0.0097	86	-0.13573
172	183.9855	192.9855	0.0097	89	-0.11364
172	183.9855	192.9855	0.0097	91	-0.13433
172	183.9855	192.9855	0.0097	81	0.1567
172	183.9855	192.9855	0.0097	86	0.33435
172	183.9855	192.9855	0.0097	87	-0.16361
172	183.9855	192.9855	0.0097	89	0.27932
172	183.9855	192.9855	0.0097	91	0.19479
172	183.9855	192.9855	0.0097	86	0.10931
172	183.9855	192.9855	0.0097	91	0.23697
172	183.9855	192.9855	0.0097	90	0.11105
173	183.986	192.986	0.0164	86	-0.16444
173	183.986	192.986	0.0164	89	-0.13706
173	183.986	192.986	0.0164	81	-0.1297
173	183.986	192.986	0.0164	86	-0.27634
173	183.986	192.986	0.0164	87	0.13532
173	183.986	192.986	0.0164	89	-0.22932
173	183.986	192.986	0.0164	91	0.28984
173	183.986	192.986	0.0164	91	-0.12933

173	183.986	192.986	0.0164	86	-0.19843
173	183.986	192.986	0.0164	89	-0.16485
173	183.986	192.986	0.0164	90	0.13786
174	183.9863	192.9863	0.0211	86	-0.1386
174	183.9863	192.9863	0.0211	89	-0.11489
174	183.9863	192.9863	0.0211	86	-0.12099
174	183.9863	192.9863	0.0211	91	0.19323
174	183.9863	192.9863	0.0211	81	0.12408
174	183.9863	192.9863	0.0211	86	0.26689
174	183.9863	192.9863	0.0211	87	-0.13027
174	183.9863	192.9863	0.0211	89	0.22074
174	183.9863	192.9863	0.0211	91	-0.17188
174	183.9863	192.9863	0.0211	88	-0.17272
174	183.9863	192.9863	0.0211	90	0.3189
174	183.9863	192.9863	0.0211	88	0.11708
175	183.9864	192.9864	0.0068	86	0.19128
175	183.9864	192.9864	0.0068	89	0.1597
175	183.9864	192.9864	0.0068	91	-0.1535
175	183.9864	192.9864	0.0068	91	-0.13273
175	183.9864	192.9864	0.0068	86	-0.15854
175	183.9864	192.9864	0.0068	89	-0.13097
175	183.9864	192.9864	0.0068	88	-0.26955
175	183.9864	192.9864	0.0068	90	0.36669
175	183.9864	192.9864	0.0068	90	-0.23806
176	183.9878	192.9878	0.0001	88	0.14103
176	183.9878	192.9878	0.0001	90	0.24129
176	183.9878	192.9878	0.0001	88	0.33406
176	183.9878	192.9878	0.0001	90	0.52582
177	183.9925	192.9925	0.0033	85	0.22693
177	183.9925	192.9925	0.0033	87	0.41569
177	183.9925	192.9925	0.0033	89	0.41494
177	183.9925	192.9925	0.0033	91	0.23896
178	183.9941	192.9941	0.0026	85	-0.22651
178	183.9941	192.9941	0.0026	87	0.41679
178	183.9941	192.9941	0.0026	89	0.41511
178	183.9941	192.9941	0.0026	91	-0.24295
179	184.0267	193.0267	0.006	84	0.12344
179	184.0267	193.0267	0.006	81	0.12293
179	184.0267	193.0267	0.006	82	-0.13211
179	184.0267	193.0267	0.006	85	-0.12198
179	184.0267	193.0267	0.006	86	0.46295
179	184.0267	193.0267	0.006	87	-0.12887
179	184.0267	193.0267	0.006	89	0.30046
179	184.0267	193.0267	0.006	91	0.2474

180	184.0276	193.0276	0.0053	84	-0.11688
180	184.0276	193.0276	0.0053	86	-0.11614
180	184.0276	193.0276	0.0053	81	0.11796
180	184.0276	193.0276	0.0053	82	0.12849
180	184.0276	193.0276	0.0053	85	0.11878
180	184.0276	193.0276	0.0053	86	0.44589
180	184.0276	193.0276	0.0053	87	-0.12409
180	184.0276	193.0276	0.0053	89	0.28658
180	184.0276	193.0276	0.0053	91	-0.24283
180	184.0276	193.0276	0.0053	86	-0.10518
181	184.0276	193.0276	0.0055	81	0.12114
181	184.0276	193.0276	0.0055	82	-0.13235
181	184.0276	193.0276	0.0055	85	-0.12131
181	184.0276	193.0276	0.0055	86	0.45529
181	184.0276	193.0276	0.0055	87	-0.12683
181	184.0276	193.0276	0.0055	89	0.29574
181	184.0276	193.0276	0.0055	91	0.24759
181	184.0276	193.0276	0.0055	86	0.1304
181	184.0276	193.0276	0.0055	84	0.10927
182	184.0285	193.0285	0.0035	81	0.12487
182	184.0285	193.0285	0.0035	82	0.13859
182	184.0285	193.0285	0.0035	85	0.12702
182	184.0285	193.0285	0.0035	86	0.47135
182	184.0285	193.0285	0.0035	87	-0.13122
182	184.0285	193.0285	0.0035	89	0.30314
182	184.0285	193.0285	0.0035	91	-0.26135
182	184.0285	193.0285	0.0035	84	-0.11626
183	184.117	193.117	0	89	0.22354
183	184.117	193.117	0	91	0.27276
183	184.117	193.117	0	87	-0.10227
183	184.117	193.117	0	89	0.36484
183	184.117	193.117	0	91	0.46991
184	184.1183	193.1183	0	87	0.10212
184	184.1183	193.1183	0	89	-0.36126
184	184.1183	193.1183	0	91	0.4729
184	184.1183	193.1183	0	89	0.22132
184	184.1183	193.1183	0	91	-0.27449
185	184.1227	193.1227	0	89	0.1751
185	184.1227	193.1227	0	91	0.21586
185	184.1227	193.1227	0	87	-0.10969
185	184.1227	193.1227	0	89	0.39069
185	184.1227	193.1227	0	91	0.49867
186	184.1239	193.1239	0	87	0.10952
186	184.1239	193.1239	0	89	-0.38685

186	184.1239	193.1239	0	91	0.50183
186	184.1239	193.1239	0	89	0.17337
186	184.1239	193.1239	0	91	-0.21722
187	184.1494	193.1494	0.0033	80	0.24888
187	184.1494	193.1494	0.0033	83	0.57273
187	184.1494	193.1494	0.0033	84	0.15145
187	184.1494	193.1494	0.0033	88	-0.20595
187	184.1494	193.1494	0.0033	90	0.19755
188	184.1511	193.1511	0.0035	80	-0.24867
188	184.1511	193.1511	0.0035	83	0.57335
188	184.1511	193.1511	0.0035	84	0.15122
188	184.1511	193.1511	0.0035	88	-0.20355
188	184.1511	193.1511	0.0035	90	-0.19869
189	184.1673	193.1673	0.0006	86	0.12858
189	184.1673	193.1673	0.0006	83	0.17438
189	184.1673	193.1673	0.0006	84	0.20644
189	184.1673	193.1673	0.0006	88	0.46313
189	184.1673	193.1673	0.0006	90	-0.33382
189	184.1673	193.1673	0.0006	88	-0.13564
190	184.1681	193.1681	0.0004	86	-0.13572
190	184.1681	193.1681	0.0004	88	0.13502
190	184.1681	193.1681	0.0004	83	0.1739
190	184.1681	193.1681	0.0004	84	0.20587
190	184.1681	193.1681	0.0004	88	0.46113
190	184.1681	193.1681	0.0004	90	0.3371
191	184.1686	193.1686	0.0033	83	0.18152
191	184.1686	193.1686	0.0033	84	0.21498
191	184.1686	193.1686	0.0033	88	0.48165
191	184.1686	193.1686	0.0033	90	-0.34065
191	184.1686	193.1686	0.0033	86	0.1249
192	184.1693	193.1693	0.0034	83	0.18097
192	184.1693	193.1693	0.0034	84	0.21433
192	184.1693	193.1693	0.0034	88	0.47971
192	184.1693	193.1693	0.0034	90	0.34478
192	184.1693	193.1693	0.0034	86	-0.132
193	184.2184	193.2184	0.0001	88	-0.38432
193	184.2184	193.2184	0.0001	90	0.58141
194	184.2196	193.2196	0.0001	88	0.37958
194	184.2196	193.2196	0.0001	90	0.58458
195	184.2276	193.2276	0.0002	88	0.11028
195	184.2276	193.2276	0.0002	90	-0.12908
195	184.2276	193.2276	0.0002	88	-0.37293
195	184.2276	193.2276	0.0002	90	0.57391
196	184.2301	193.2301	0.0002	88	0.36879

196	184.2301	193.2301	0.0002	90	0.57691
196	184.2301	193.2301	0.0002	88	0.1088
196	184.2301	193.2301	0.0002	90	0.1296
197	184.2339	193.2339	0.001	81	0.44512
197	184.2339	193.2339	0.001	82	0.44492
197	184.2339	193.2339	0.001	85	0.23402
197	184.2339	193.2339	0.001	86	-0.15608
197	184.2339	193.2339	0.001	87	0.12532
198	184.2346	193.2346	0.001	81	-0.44478
198	184.2346	193.2346	0.001	82	0.44478
198	184.2346	193.2346	0.001	85	0.23447
198	184.2346	193.2346	0.001	86	0.15633
198	184.2346	193.2346	0.001	87	-0.12576
199	184.2403	193.2403	0.0065	82	-0.10704
199	184.2403	193.2403	0.0065	86	-0.14475
199	184.2403	193.2403	0.0065	91	-0.11261
199	184.2403	193.2403	0.0065	88	-0.26896
199	184.2403	193.2403	0.0065	90	0.20329
199	184.2403	193.2403	0.0065	84	0.1328
199	184.2403	193.2403	0.0065	88	0.42353
199	184.2403	193.2403	0.0065	90	-0.2729
200	184.2416	193.2416	0.0005	88	0.12808
200	184.2416	193.2416	0.0005	90	-0.16551
200	184.2416	193.2416	0.0005	83	0.10211
200	184.2416	193.2416	0.0005	84	0.15127
200	184.2416	193.2416	0.0005	88	0.48223
200	184.2416	193.2416	0.0005	90	-0.29649
200	184.2416	193.2416	0.0005	86	-0.14056
201	184.2417	193.2417	0.0056	82	-0.108
201	184.2417	193.2417	0.0056	86	0.14971
201	184.2417	193.2417	0.0056	87	0.10036
201	184.2417	193.2417	0.0056	91	-0.1137
201	184.2417	193.2417	0.0056	84	0.13136
201	184.2417	193.2417	0.0056	88	0.41776
201	184.2417	193.2417	0.0056	90	0.2739
201	184.2417	193.2417	0.0056	88	0.27214
201	184.2417	193.2417	0.0056	90	0.20852
202	184.243	193.243	0.0003	83	0.10235
202	184.243	193.243	0.0003	84	0.15182
202	184.243	193.243	0.0003	88	0.48367
202	184.243	193.243	0.0003	90	0.30099
202	184.243	193.243	0.0003	88	-0.1231
202	184.243	193.243	0.0003	90	-0.16412
202	184.243	193.243	0.0003	86	0.14279

203	184.3207	193.3207	0.0031	90	0.11813
203	184.3207	193.3207	0.0031	85	0.15716
203	184.3207	193.3207	0.0031	86	0.37303
203	184.3207	193.3207	0.0031	87	0.40674
203	184.3207	193.3207	0.0031	89	-0.1869
203	184.3207	193.3207	0.0031	91	-0.21818
204	184.3211	193.3211	0.0037	90	0.11269
204	184.3211	193.3211	0.0037	85	-0.15689
204	184.3211	193.3211	0.0037	86	0.37309
204	184.3211	193.3211	0.0037	87	0.40729
204	184.3211	193.3211	0.0037	89	-0.18542
204	184.3211	193.3211	0.0037	91	0.2203
205	184.3216	193.3216	0.0005	85	0.16372
205	184.3216	193.3216	0.0005	86	0.3804
205	184.3216	193.3216	0.0005	87	0.41431
205	184.3216	193.3216	0.0005	89	-0.18954
205	184.3216	193.3216	0.0005	91	-0.22754
205	184.3216	193.3216	0.0005	90	0.11355
206	184.322	193.322	0.0005	85	-0.16333
206	184.322	193.322	0.0005	86	0.38034
206	184.322	193.322	0.0005	87	0.41451
206	184.322	193.322	0.0005	89	-0.18782
206	184.322	193.322	0.0005	91	0.22951
206	184.322	193.322	0.0005	90	0.10768
207	184.3422	193.3422	0.0045	90	-0.11472
207	184.3422	193.3422	0.0045	88	0.15822
207	184.3422	193.3422	0.0045	90	-0.13439
207	184.3422	193.3422	0.0045	86	-0.15742
207	184.3422	193.3422	0.0045	91	-0.17275
207	184.3422	193.3422	0.0045	86	0.49113
207	184.3422	193.3422	0.0045	87	0.2589
207	184.3422	193.3422	0.0045	89	-0.15222
207	184.3422	193.3422	0.0045	91	0.13362
208	184.3428	193.3428	0.0013	88	-0.16078
208	184.3428	193.3428	0.0013	90	-0.14147
208	184.3428	193.3428	0.0013	90	0.10476
208	184.3428	193.3428	0.0013	86	0.49109
208	184.3428	193.3428	0.0013	87	0.25702
208	184.3428	193.3428	0.0013	89	-0.15271
208	184.3428	193.3428	0.0013	91	-0.13064
208	184.3428	193.3428	0.0013	86	0.15698
208	184.3428	193.3428	0.0013	91	-0.16941
209	184.3432	193.3432	0.0001	91	0.127
209	184.3432	193.3432	0.0001	86	0.51185

209	184.3432	193.3432	0.0001	87	0.26643
209	184.3432	193.3432	0.0001	89	-0.15978
209	184.3432	193.3432	0.0001	91	0.17526
209	184.3432	193.3432	0.0001	90	0.12431
209	184.3432	193.3432	0.0001	88	0.14378
209	184.3432	193.3432	0.0001	90	-0.11227
210	184.3436	193.3436	0.0001	86	0.51248
210	184.3436	193.3436	0.0001	87	0.26662
210	184.3436	193.3436	0.0001	89	-0.16104
210	184.3436	193.3436	0.0001	91	-0.17479
210	184.3436	193.3436	0.0001	91	0.12943
210	184.3436	193.3436	0.0001	88	-0.1475
210	184.3436	193.3436	0.0001	90	-0.11566
210	184.3436	193.3436	0.0001	90	-0.13661
211	184.3503	193.3503	0.0001	87	-0.11774
211	184.3503	193.3503	0.0001	89	0.41436
211	184.3503	193.3503	0.0001	91	0.55301
212	184.3518	193.3518	0.0001	87	0.11746
212	184.3518	193.3518	0.0001	89	-0.40996
212	184.3518	193.3518	0.0001	91	0.55629
213	184.3857	193.3857	0.0001	87	-0.12892
213	184.3857	193.3857	0.0001	89	0.43186
213	184.3857	193.3857	0.0001	91	0.50961
213	184.3857	193.3857	0.0001	91	0.1643
214	184.3863	193.3863	0.0002	91	-0.16534
214	184.3863	193.3863	0.0002	87	0.12865
214	184.3863	193.3863	0.0002	89	-0.42776
214	184.3863	193.3863	0.0002	91	0.51296
215	184.408	193.408	0.0159	85	0.26926
215	184.408	193.408	0.0159	87	0.20071
215	184.408	193.408	0.0159	89	0.10791
215	184.408	193.408	0.0159	91	0.15476
215	184.408	193.408	0.0159	86	-0.10168
215	184.408	193.408	0.0159	87	0.50018
215	184.408	193.408	0.0159	89	0.26913
216	184.4087	193.4087	0.0001	80	-0.28329
216	184.4087	193.4087	0.0001	83	-0.13228
216	184.4087	193.4087	0.0001	84	0.59512
216	184.4087	193.4087	0.0001	88	-0.15041
216	184.4087	193.4087	0.0001	90	0.11791
217	184.4087	193.4087	0.0007	87	0.49502
217	184.4087	193.4087	0.0007	89	0.26509
217	184.4087	193.4087	0.0007	85	0.26924
217	184.4087	193.4087	0.0007	87	-0.20224

217	184.4087	193.4087	0.0007	89	-0.10842
217	184.4087	193.4087	0.0007	91	0.1559
218	184.4089	193.4089	0	85	0.22951
218	184.4089	193.4089	0	86	-0.10388
218	184.4089	193.4089	0	87	0.52323
218	184.4089	193.4089	0	89	0.27908
218	184.4089	193.4089	0	91	0.12967
218	184.4089	193.4089	0	85	-0.13845
218	184.4089	193.4089	0	87	-0.10795
219	184.4092	193.4092	0	85	-0.1406
219	184.4092	193.4092	0	87	0.11151
219	184.4092	193.4092	0	85	-0.22839
219	184.4092	193.4092	0	86	-0.10392
219	184.4092	193.4092	0	87	0.52385
219	184.4092	193.4092	0	89	0.27918
219	184.4092	193.4092	0	91	-0.13179
220	184.4098	193.4098	0.0001	80	0.28462
220	184.4098	193.4098	0.0001	83	-0.13263
220	184.4098	193.4098	0.0001	84	0.59988
220	184.4098	193.4098	0.0001	88	-0.15006
220	184.4098	193.4098	0.0001	90	-0.1196
221	184.4426	193.4426	0.003	85	0.29442
221	184.4426	193.4426	0.003	86	-0.1963
221	184.4426	193.4426	0.003	87	0.37331
221	184.4426	193.4426	0.003	89	0.38776
221	184.4426	193.4426	0.003	91	0.19471
222	184.4435	193.4435	0.0035	85	-0.29398
222	184.4435	193.4435	0.0035	86	-0.19603
222	184.4435	193.4435	0.0035	87	0.37309
222	184.4435	193.4435	0.0035	89	0.38693
222	184.4435	193.4435	0.0035	91	-0.19775
223	184.4438	193.4438	0.0003	85	0.30622
223	184.4438	193.4438	0.0003	86	-0.20016
223	184.4438	193.4438	0.0003	87	0.37957
223	184.4438	193.4438	0.0003	89	0.39491
223	184.4438	193.4438	0.0003	91	0.20208
224	184.4448	193.4448	0.0002	85	-0.30636
224	184.4448	193.4448	0.0002	86	-0.19993
224	184.4448	193.4448	0.0002	87	0.37958
224	184.4448	193.4448	0.0002	89	0.39399
224	184.4448	193.4448	0.0002	91	-0.20548
225	184.6588	193.6588	0	89	-0.14013
225	184.6588	193.6588	0	88	-0.34509
225	184.6588	193.6588	0	90	0.42258

225	184.6588	193.6588	0	88	-0.20583
225	184.6588	193.6588	0	90	0.3398
226	184.6597	193.6597	0	88	-0.37357
226	184.6597	193.6597	0	90	0.49773
226	184.6597	193.6597	0	88	0.14569
226	184.6597	193.6597	0	90	-0.2087
226	184.6597	193.6597	0	89	-0.12154
227	184.6601	193.6601	0	89	-0.13536
227	184.6601	193.6601	0	88	-0.20489
227	184.6601	193.6601	0	90	-0.34506
227	184.6601	193.6601	0	88	0.33767
227	184.6601	193.6601	0	90	0.41565
228	184.6611	193.6611	0	88	0.14224
228	184.6611	193.6611	0	90	0.20034
228	184.6611	193.6611	0	88	0.36627
228	184.6611	193.6611	0	90	0.5014
228	184.6611	193.6611	0	89	-0.11887
229	184.6931	193.6931	0.0003	87	-0.10653
229	184.6931	193.6931	0.0003	88	-0.10385
229	184.6931	193.6931	0.0003	90	0.12331
229	184.6931	193.6931	0.0003	88	-0.38561
229	184.6931	193.6931	0.0003	90	0.50191
229	184.6931	193.6931	0.0003	92	-0.13722
230	184.6945	193.6945	0.0008	88	-0.39697
230	184.6945	193.6945	0.0008	90	0.51304
230	184.6945	193.6945	0.0008	92	-0.14162
230	184.6945	193.6945	0.0008	87	-0.10206
231	184.6946	193.6946	0.0003	87	-0.10469
231	184.6946	193.6946	0.0003	88	0.38134
231	184.6946	193.6946	0.0003	90	0.50447
231	184.6946	193.6946	0.0003	92	0.13692
231	184.6946	193.6946	0.0003	88	-0.10326
231	184.6946	193.6946	0.0003	90	-0.12143
232	184.6961	193.6961	0.0008	88	0.39302
232	184.6961	193.6961	0.0008	90	0.51702
232	184.6961	193.6961	0.0008	92	0.1414
232	184.6961	193.6961	0.0008	87	-0.1006
233	184.7013	193.7013	0.0003	82	0.14165
233	184.7013	193.7013	0.0003	85	0.16319
233	184.7013	193.7013	0.0003	86	0.54428
233	184.7013	193.7013	0.0003	87	-0.13292
233	184.7013	193.7013	0.0003	89	0.27348
233	184.7013	193.7013	0.0003	91	-0.22262
234	184.7015	193.7015	0.0004	82	-0.14193

234	184.7015	193.7015	0.0004	85	-0.16316
234	184.7015	193.7015	0.0004	86	0.5439
234	184.7015	193.7015	0.0004	87	-0.13296
234	184.7015	193.7015	0.0004	89	0.27608
234	184.7015	193.7015	0.0004	91	0.22102
235	184.7199	193.7199	0.0024	92	0.43104
235	184.7199	193.7199	0.0024	93	-0.40274
235	184.7199	193.7199	0.0024	92	-0.28238
235	184.7199	193.7199	0.0024	93	0.21758
236	184.7203	193.7203	0.0029	92	0.28027
236	184.7203	193.7203	0.0029	93	0.21986
236	184.7203	193.7203	0.0029	92	0.42755
236	184.7203	193.7203	0.0029	93	0.40663
237	184.7249	193.7249	0.0001	92	0.46917
237	184.7249	193.7249	0.0001	93	-0.42088
237	184.7249	193.7249	0.0001	92	-0.21244
237	184.7249	193.7249	0.0001	93	0.18031
238	184.7253	193.7253	0.0001	92	0.21072
238	184.7253	193.7253	0.0001	93	0.18203
238	184.7253	193.7253	0.0001	92	0.46548
238	184.7253	193.7253	0.0001	93	0.42503
239	184.7856	193.7856	0.0012	83	0.2463
239	184.7856	193.7856	0.0012	84	0.24834
239	184.7856	193.7856	0.0012	88	0.48805
239	184.7856	193.7856	0.0012	90	-0.36177
240	184.7875	193.7875	0.0013	83	0.24579
240	184.7875	193.7875	0.0013	84	0.24732
240	184.7875	193.7875	0.0013	88	0.48578
240	184.7875	193.7875	0.0013	90	0.36579
241	184.8193	193.8193	0.0003	89	-0.26918
241	184.8193	193.8193	0.0003	91	-0.27682
241	184.8193	193.8193	0.0003	89	0.37729
241	184.8193	193.8193	0.0003	91	0.40907
242	184.8202	193.8202	0	89	0.17365
242	184.8202	193.8202	0	91	0.18233
242	184.8202	193.8202	0	87	-0.10496
242	184.8202	193.8202	0	89	0.4289
242	184.8202	193.8202	0	91	0.4571
243	184.8206	193.8206	0.0003	89	-0.37397
243	184.8206	193.8206	0.0003	91	0.41122
243	184.8206	193.8206	0.0003	89	-0.26861
243	184.8206	193.8206	0.0003	91	0.28063
244	184.8215	193.8215	0	87	0.1053
244	184.8215	193.8215	0	89	-0.42743

244	184.8215	193.8215	0	91	0.46265
244	184.8215	193.8215	0	89	0.17117
244	184.8215	193.8215	0	91	-0.18255
245	184.8673	193.8673	0.0009	90	-0.11673
245	184.8673	193.8673	0.0009	87	-0.12637
245	184.8673	193.8673	0.0009	89	0.38792
245	184.8673	193.8673	0.0009	91	0.50813
245	184.8673	193.8673	0.0009	91	-0.15
246	184.8679	193.8679	0.0008	90	0.12191
246	184.8679	193.8679	0.0008	91	0.15087
246	184.8679	193.8679	0.0008	87	0.12659
246	184.8679	193.8679	0.0008	89	-0.38434
246	184.8679	193.8679	0.0008	91	0.51188
247	184.8686	193.8686	0.0001	87	-0.1295
247	184.8686	193.8686	0.0001	89	0.3954
247	184.8686	193.8686	0.0001	91	0.52805
247	184.8686	193.8686	0.0001	90	-0.11213
248	184.8691	193.8691	0	87	0.12973
248	184.8691	193.8691	0	89	-0.39168
248	184.8691	193.8691	0	91	0.53161
248	184.8691	193.8691	0	90	0.11725
249	184.9039	193.9039	0.0019	88	-0.11766
249	184.9039	193.9039	0.0019	92	0.55919
249	184.9039	193.9039	0.0019	93	-0.39852
250	184.9041	193.9041	0.0021	88	-0.11772
250	184.9041	193.9041	0.0021	92	0.55564
250	184.9041	193.9041	0.0021	93	0.40324
251	184.9811	193.9811	0.0003	85	-0.15901
251	184.9811	193.9811	0.0003	86	0.34836
251	184.9811	193.9811	0.0003	87	0.50857
251	184.9811	193.9811	0.0003	89	-0.20158
251	184.9811	193.9811	0.0003	91	0.20673
252	184.9815	193.9815	0.0004	85	0.15905
252	184.9815	193.9815	0.0004	86	0.34905
252	184.9815	193.9815	0.0004	87	0.50804
252	184.9815	193.9815	0.0004	89	-0.20337
252	184.9815	193.9815	0.0004	91	-0.20506

Table S4. Tabulated TDDFT data and transitions from B 1s orbitals for **1**. An energy shift of +9.0 eV was applied to the calculated spectra so that relative differences in calculated and experimental peak positions could be compared.

Excited State	Energy (eV)	Shifted Energy (eV)	Oscillator Strength (f-value)	To Orbital	Coefficient

1	178.2182	187.2182	0.0016	78	0.21996
1	178.2182	187.2182	0.0016	78	0.66895
2	178.2186	187.2186	0.0007	78	0.66894
2	178.2186	187.2186	0.0007	78	-0.21995
3	178.2243	187.2243	0.0053	78	0.15947
3	178.2243	187.2243	0.0053	78	0.68102
4	178.2246	187.2246	0.0025	78	0.681
4	178.2246	187.2246	0.0025	78	-0.15926
5	178.2737	187.2737	0.0098	78	-0.16928
5	178.2737	187.2737	0.0098	78	0.68035
6	178.274	187.274	0.0102	78	0.68042
6	178.274	187.274	0.0102	78	0.16945
7	178.3372	187.3372	0.0032	79	-0.35131
7	178.3372	187.3372	0.0032	79	0.60913
8	178.3373	187.3373	0.0003	79	0.60913
8	178.3373	187.3373	0.0003	79	0.35133
9	178.3421	187.3421	0.003	79	0.5335
9	178.3421	187.3421	0.003	79	-0.45944
10	178.3422	187.3422	0.0008	79	0.45944
10	178.3422	187.3422	0.0008	79	0.53352
11	178.5078	187.5078	0.0023	79	0.70583
12	178.5089	187.5089	0.0069	79	0.70584
13	178.5118	187.5118	0.0031	78	0.70568
14	178.5122	187.5122	0.0031	78	0.70568
15	178.576	187.576	0.0038	79	0.70533
16	178.5761	187.5761	0.0037	79	0.70533
17	178.7998	187.7998	0.0021	78	0.56817
17	178.7998	187.7998	0.0021	78	0.2054
17	178.7998	187.7998	0.0021	79	0.33862
18	178.8002	187.8002	0.008	78	0.45501
18	178.8002	187.8002	0.008	79	-0.16656
18	178.8002	187.8002	0.008	79	-0.29051
18	178.8002	187.8002	0.008	78	-0.41416
19	178.8004	187.8004	0.0137	78	0.28923
19	178.8004	187.8004	0.0137	79	0.14454
19	178.8004	187.8004	0.0137	78	0.2164
19	178.8004	187.8004	0.0137	79	0.27102
19	178.8004	187.8004	0.0137	78	-0.35371
19	178.8004	187.8004	0.0137	79	-0.14335
19	178.8004	187.8004	0.0137	78	0.35436
20	178.8008	187.8008	0.0113	78	0.28892
20	178.8008	187.8008	0.0113	79	-0.3022
20	178.8008	187.8008	0.0113	79	0.10708
20	178.8008	187.8008	0.0113	78	0.46017

20	178.8008	187.8008	0.0113	79	-0.1055
20	178.8008	187.8008	0.0113	78	0.27866
21	178.9227	187.9227	0	78	0.11213
21	178.9227	187.9227	0	78	-0.31928
21	178.9227	187.9227	0	79	0.61078
22	178.9234	187.9234	0.0023	78	0.3209
22	178.9234	187.9234	0.0023	79	0.10676
22	178.9234	187.9234	0.0023	79	0.60318
23	178.9239	187.9239	0.0073	79	0.31072
23	178.9239	187.9239	0.0073	79	0.53048
23	178.9239	187.9239	0.0073	78	0.22657
23	178.9239	187.9239	0.0073	78	-0.22867
24	178.9239	187.9239	0.0038	79	0.52112
24	178.9239	187.9239	0.0038	79	-0.31722
24	178.9239	187.9239	0.0038	78	0.24186
24	178.9239	187.9239	0.0038	78	0.23745
25	179.0394	188.0394	0.0041	79	0.18162
25	179.0394	188.0394	0.0041	78	0.66321
26	179.0396	188.0396	0.001	79	-0.16288
26	179.0396	188.0396	0.001	78	0.65998
26	179.0396	188.0396	0.001	79	0.12594
27	179.0403	188.0403	0.0008	78	0.64899
27	179.0403	188.0403	0.0008	79	0.12867
27	179.0403	188.0403	0.0008	79	0.2318
28	179.0407	188.0407	0.0009	78	0.64925
28	179.0407	188.0407	0.0009	79	-0.14561
28	179.0407	188.0407	0.0009	79	-0.21081
29	179.0531	188.0531	0.001	78	-0.23618
29	179.0531	188.0531	0.001	79	0.10486
29	179.0531	188.0531	0.001	78	0.11959
29	179.0531	188.0531	0.001	79	0.64026
30	179.0537	188.0537	0.001	78	0.23321
30	179.0537	188.0537	0.001	78	-0.11868
30	179.0537	188.0537	0.001	79	0.64354
30	179.0537	188.0537	0.001	79	-0.105
31	179.0546	188.0546	0.0065	78	-0.13846
31	179.0546	188.0546	0.0065	79	0.65907
31	179.0546	188.0546	0.0065	78	-0.18903
32	179.055	188.055	0.0058	78	0.12759
32	179.055	188.055	0.0058	79	0.66294
32	179.055	188.055	0.0058	78	0.1877
33	179.6062	188.6062	0.0049	78	0.70563
34	179.607	188.607	0.004	78	0.70563
35	179.7314	188.7314	0.0003	79	0.70615

36	179.7319	188.7319	0.0002	79	0.70615
37	182.013	191.013	0.032	80	0.4645
37	182.013	191.013	0.032	83	0.23654
37	182.013	191.013	0.032	84	-0.22194
37	182.013	191.013	0.032	80	-0.39465
38	182.0133	191.0133	0.0162	80	0.39451
38	182.0133	191.0133	0.0162	80	0.46438
38	182.0133	191.0133	0.0162	83	-0.237
38	182.0133	191.0133	0.0162	84	0.22219
39	182.0147	191.0147	0.002	80	0.56004
39	182.0147	191.0147	0.002	83	0.23148
39	182.0147	191.0147	0.002	84	-0.21725
39	182.0147	191.0147	0.002	80	-0.2389
39	182.0147	191.0147	0.002	83	-0.10527
40	182.015	191.015	0.0021	80	0.23876
40	182.015	191.015	0.0021	83	-0.10545
40	182.015	191.015	0.0021	80	0.55992
40	182.015	191.015	0.0021	83	-0.23201
40	182.015	191.015	0.0021	84	0.21754
41	182.2261	191.2261	0.0023	80	0.60551
41	182.2261	191.2261	0.0023	83	0.28241
41	182.2261	191.2261	0.0023	84	-0.22024
42	182.2263	191.2263	0.0023	80	0.60518
42	182.2263	191.2263	0.0023	83	-0.28294
42	182.2263	191.2263	0.0023	84	0.22048
43	182.4593	191.4593	0.0031	80	0.6362
43	182.4593	191.4593	0.0031	83	0.21159
43	182.4593	191.4593	0.0031	84	-0.16851
44	182.4605	191.4605	0.0064	80	0.63593
44	182.4605	191.4605	0.0064	83	-0.21211
44	182.4605	191.4605	0.0064	84	0.16866
45	182.5449	191.5449	0.0065	82	0.24658
45	182.5449	191.5449	0.0065	85	-0.13392
45	182.5449	191.5449	0.0065	87	-0.13776
45	182.5449	191.5449	0.0065	82	0.5154
45	182.5449	191.5449	0.0065	85	-0.27946
45	182.5449	191.5449	0.0065	87	-0.18939
46	182.5452	191.5452	0.0045	82	0.51562
46	182.5452	191.5452	0.0045	85	-0.27898
46	182.5452	191.5452	0.0045	87	0.18938
46	182.5452	191.5452	0.0045	82	-0.24694
46	182.5452	191.5452	0.0045	85	0.13339
46	182.5452	191.5452	0.0045	87	-0.13791
47	182.5524	191.5524	0.001	82	0.18054

47	182.5524	191.5524	0.001	87	-0.1129
47	182.5524	191.5524	0.001	82	0.54641
47	182.5524	191.5524	0.001	85	-0.28885
47	182.5524	191.5524	0.001	87	-0.20156
48	182.5527	191.5527	0.0007	82	0.54673
48	182.5527	191.5527	0.0007	85	-0.28822
48	182.5527	191.5527	0.0007	87	0.2016
48	182.5527	191.5527	0.0007	82	-0.18074
48	182.5527	191.5527	0.0007	87	-0.11295
49	182.6835	191.6835	0	81	-0.33636
49	182.6835	191.6835	0	82	-0.11306
49	182.6835	191.6835	0	85	-0.28199
49	182.6835	191.6835	0	81	0.51236
50	182.6836	191.6836	0	81	0.51131
50	182.6836	191.6836	0	81	0.3359
50	182.6836	191.6836	0	82	-0.11439
50	182.6836	191.6836	0	85	-0.28364
51	182.6893	191.6893	0.0003	81	0.46586
51	182.6893	191.6893	0.0003	82	0.10678
51	182.6893	191.6893	0.0003	85	0.27773
51	182.6893	191.6893	0.0003	81	-0.40186
52	182.6893	191.6893	0.001	81	0.40115
52	182.6893	191.6893	0.001	81	0.46545
52	182.6893	191.6893	0.001	82	-0.10778
52	182.6893	191.6893	0.001	85	-0.27891
53	182.7104	191.7104	0.0034	81	0.15751
53	182.7104	191.7104	0.0034	82	0.17056
53	182.7104	191.7104	0.0034	85	0.22745
53	182.7104	191.7104	0.0034	86	-0.11769
53	182.7104	191.7104	0.0034	81	0.26651
53	182.7104	191.7104	0.0034	82	0.28065
53	182.7104	191.7104	0.0034	85	0.3743
53	182.7104	191.7104	0.0034	86	-0.20096
53	182.7104	191.7104	0.0034	87	0.16141
54	182.7107	191.7107	0.0035	81	-0.26659
54	182.7107	191.7107	0.0035	82	0.28061
54	182.7107	191.7107	0.0035	85	0.37533
54	182.7107	191.7107	0.0035	86	0.20091
54	182.7107	191.7107	0.0035	87	-0.16183
54	182.7107	191.7107	0.0035	81	0.15519
54	182.7107	191.7107	0.0035	82	-0.16981
54	182.7107	191.7107	0.0035	85	-0.22715
54	182.7107	191.7107	0.0035	86	-0.11802
55	182.7184	191.7184	0.0057	81	0.14187

55	182.7184	191.7184	0.0057	82	0.10963
55	182.7184	191.7184	0.0057	85	0.15177
55	182.7184	191.7184	0.0057	86	-0.10749
55	182.7184	191.7184	0.0057	81	0.26949
55	182.7184	191.7184	0.0057	82	0.30257
55	182.7184	191.7184	0.0057	85	0.4188
55	182.7184	191.7184	0.0057	86	-0.20525
55	182.7184	191.7184	0.0057	87	0.16844
56	182.7188	191.7188	0.0043	81	-0.26896
56	182.7188	191.7188	0.0043	82	0.30225
56	182.7188	191.7188	0.0043	85	0.41963
56	182.7188	191.7188	0.0043	86	0.2053
56	182.7188	191.7188	0.0043	87	-0.16896
56	182.7188	191.7188	0.0043	81	0.14027
56	182.7188	191.7188	0.0043	82	-0.10923
56	182.7188	191.7188	0.0043	85	-0.15162
56	182.7188	191.7188	0.0043	86	-0.10763
57	182.7386	191.7386	0.0054	80	0.14372
57	182.7386	191.7386	0.0054	80	0.5835
57	182.7386	191.7386	0.0054	83	0.24377
57	182.7386	191.7386	0.0054	84	-0.22837
58	182.7393	191.7393	0.0053	80	0.58353
58	182.7393	191.7393	0.0053	83	-0.24434
58	182.7393	191.7393	0.0053	84	0.22876
58	182.7393	191.7393	0.0053	80	-0.14599
59	182.7406	191.7406	0.0088	80	0.60023
59	182.7406	191.7406	0.0088	83	0.25159
59	182.7406	191.7406	0.0088	84	-0.23535
60	182.7414	191.7414	0.0089	80	0.6008
60	182.7414	191.7414	0.0089	83	-0.25242
60	182.7414	191.7414	0.0089	84	0.23596
61	182.7593	191.7593	0.0006	81	-0.35396
61	182.7593	191.7593	0.0006	82	0.53468
61	182.7593	191.7593	0.0006	85	-0.19909
61	182.7593	191.7593	0.0006	87	-0.18942
62	182.7598	191.7598	0.0006	81	0.35536
62	182.7598	191.7598	0.0006	82	0.53473
62	182.7598	191.7598	0.0006	85	-0.19708
62	182.7598	191.7598	0.0006	87	0.18872
63	182.8047	191.8047	0.0078	82	-0.15343
63	182.8047	191.8047	0.0078	81	-0.26345
63	182.8047	191.8047	0.0078	82	0.5683
63	182.8047	191.8047	0.0078	85	-0.19132
63	182.8047	191.8047	0.0078	87	0.15959

64	182.8054	191.8054	0.0075	81	0.26304
64	182.8054	191.8054	0.0075	82	0.56826
64	182.8054	191.8054	0.0075	85	-0.19184
64	182.8054	191.8054	0.0075	87	-0.15929
64	182.8054	191.8054	0.0075	82	0.1534
65	182.8287	191.8287	0.002	81	-0.40256
65	182.8287	191.8287	0.002	85	0.49839
65	182.8287	191.8287	0.002	87	0.22694
65	182.8287	191.8287	0.002	89	0.14821
66	182.829	191.829	0.002	81	0.40188
66	182.829	191.829	0.002	85	0.49858
66	182.829	191.829	0.002	87	-0.22785
66	182.829	191.829	0.002	89	-0.14876
67	182.8379	191.8379	0.0021	80	0.10406
67	182.8379	191.8379	0.0021	80	0.10733
67	182.8379	191.8379	0.0021	83	0.30896
67	182.8379	191.8379	0.0021	80	0.178
67	182.8379	191.8379	0.0021	83	0.5241
67	182.8379	191.8379	0.0021	88	-0.12882
67	182.8379	191.8379	0.0021	90	0.10038
68	182.8385	191.8385	0.0025	80	0.14896
68	182.8385	191.8385	0.0025	80	-0.17766
68	182.8385	191.8385	0.0025	83	0.50984
68	182.8385	191.8385	0.0025	88	-0.12418
68	182.8385	191.8385	0.0025	90	-0.10134
68	182.8385	191.8385	0.0025	80	0.10151
68	182.8385	191.8385	0.0025	83	-0.31737
69	182.839	191.839	0.0001	80	0.10931
69	182.839	191.839	0.0001	83	0.2068
69	182.839	191.839	0.0001	84	-0.20806
69	182.839	191.839	0.0001	80	0.57567
69	182.839	191.839	0.0001	83	0.18068
70	182.8399	191.8399	0.0046	80	0.56955
70	182.8399	191.8399	0.0046	83	0.20625
70	182.8399	191.8399	0.0046	84	-0.20721
70	182.8399	191.8399	0.0046	83	-0.18579
71	182.8418	191.8418	0.0359	80	0.54965
71	182.8418	191.8418	0.0359	80	0.22243
71	182.8418	191.8418	0.0359	83	-0.1934
71	182.8418	191.8418	0.0359	84	0.19459
71	182.8418	191.8418	0.0359	83	-0.1697
72	182.8428	191.8428	0.0027	80	-0.17977
72	182.8428	191.8428	0.0027	83	-0.15123
72	182.8428	191.8428	0.0027	84	0.15213

72	182.8428	191.8428	0.0027	80	0.40565
72	182.8428	191.8428	0.0027	80	0.13743
72	182.8428	191.8428	0.0027	80	-0.12065
72	182.8428	191.8428	0.0027	83	0.16413
72	182.8428	191.8428	0.0027	80	0.12068
72	182.8428	191.8428	0.0027	83	0.34691
73	182.8428	191.8428	0.0034	80	0.13226
73	182.8428	191.8428	0.0034	83	0.12716
73	182.8428	191.8428	0.0034	84	-0.12795
73	182.8428	191.8428	0.0034	80	-0.38599
73	182.8428	191.8428	0.0034	80	-0.12684
73	182.8428	191.8428	0.0034	83	0.12485
73	182.8428	191.8428	0.0034	80	0.12866
73	182.8428	191.8428	0.0034	83	0.42477
73	182.8428	191.8428	0.0034	88	-0.10455
74	182.8434	191.8434	0.0069	80	0.15246
74	182.8434	191.8434	0.0069	80	-0.18018
74	182.8434	191.8434	0.0069	83	0.55582
74	182.8434	191.8434	0.0069	88	-0.1356
74	182.8434	191.8434	0.0069	90	-0.10364
74	182.8434	191.8434	0.0069	83	-0.21245
75	183.003	192.003	0.0081	81	-0.17948
75	183.003	192.003	0.0081	85	-0.15368
75	183.003	192.003	0.0081	80	-0.14611
75	183.003	192.003	0.0081	84	0.26379
75	183.003	192.003	0.0081	80	-0.21753
75	183.003	192.003	0.0081	84	0.50172
75	183.003	192.003	0.0081	88	-0.12983
76	183.0033	192.0033	0.0105	81	-0.21492
76	183.0033	192.0033	0.0105	85	0.17742
76	183.0033	192.0033	0.0105	80	0.21213
76	183.0033	192.0033	0.0105	84	0.48293
76	183.0033	192.0033	0.0105	88	-0.12413
76	183.0033	192.0033	0.0105	80	-0.13915
76	183.0033	192.0033	0.0105	84	-0.2608
77	183.0074	192.0074	0.0078	85	0.19794
77	183.0074	192.0074	0.0078	81	0.45278
77	183.0074	192.0074	0.0078	85	-0.32043
77	183.0074	192.0074	0.0078	86	-0.11378
77	183.0074	192.0074	0.0078	87	0.1835
77	183.0074	192.0074	0.0078	84	0.26211
78	183.0078	192.0078	0.0025	81	0.46695
78	183.0078	192.0078	0.0025	85	0.33297
78	183.0078	192.0078	0.0025	86	-0.11799

78	183.0078	192.0078	0.0025	87	0.18964
78	183.0078	192.0078	0.0025	85	0.20379
78	183.0078	192.0078	0.0025	84	0.22218
79	183.0084	192.0084	0.0004	80	-0.13112
79	183.0084	192.0084	0.0004	84	0.20693
79	183.0084	192.0084	0.0004	80	-0.24983
79	183.0084	192.0084	0.0004	84	0.57302
79	183.0084	192.0084	0.0004	88	-0.14809
80	183.0089	192.0089	0.0005	80	0.24988
80	183.0089	192.0089	0.0005	84	0.57404
80	183.0089	192.0089	0.0005	88	-0.14726
80	183.0089	192.0089	0.0005	80	-0.13115
80	183.0089	192.0089	0.0005	84	-0.20726
81	183.0163	192.0163	0.0017	81	0.4202
81	183.0163	192.0163	0.0017	82	0.40514
81	183.0163	192.0163	0.0017	85	0.27208
81	183.0163	192.0163	0.0017	86	-0.21625
81	183.0163	192.0163	0.0017	87	0.1435
81	183.0163	192.0163	0.0017	91	0.10362
82	183.0166	192.0166	0.0018	81	0.41963
82	183.0166	192.0166	0.0018	82	-0.40498
82	183.0166	192.0166	0.0018	85	-0.27274
82	183.0166	192.0166	0.0018	86	-0.21659
82	183.0166	192.0166	0.0018	87	0.14407
82	183.0166	192.0166	0.0018	91	-0.10335
83	183.022	192.022	0.0078	80	0.22003
83	183.022	192.022	0.0078	83	0.57028
83	183.022	192.022	0.0078	84	0.17939
83	183.022	192.022	0.0078	88	-0.2265
83	183.022	192.022	0.0078	90	0.18532
84	183.023	192.023	0.0083	80	-0.21972
84	183.023	192.023	0.0083	83	0.57083
84	183.023	192.023	0.0083	84	0.17949
84	183.023	192.023	0.0083	88	-0.22449
84	183.023	192.023	0.0083	90	-0.18701
85	183.0894	192.0894	0.0038	85	-0.11167
85	183.0894	192.0894	0.0038	81	0.3933
85	183.0894	192.0894	0.0038	82	0.3353
85	183.0894	192.0894	0.0038	85	0.39841
85	183.0894	192.0894	0.0038	86	0.1234
85	183.0894	192.0894	0.0038	87	-0.12219
86	183.0902	192.0902	0.0035	81	-0.39432
86	183.0902	192.0902	0.0035	82	0.33559
86	183.0902	192.0902	0.0035	85	0.39793

86	183.0902	192.0902	0.0035	86	-0.12317
86	183.0902	192.0902	0.0035	87	0.12166
86	183.0902	192.0902	0.0035	85	0.11127
87	183.1297	192.1297	0.0062	80	0.14869
87	183.1297	192.1297	0.0062	80	-0.20428
87	183.1297	192.1297	0.0062	83	0.63777
87	183.1297	192.1297	0.0062	88	-0.11264
88	183.13	192.13	0.0012	80	0.20484
88	183.13	192.13	0.0012	83	0.63753
88	183.13	192.13	0.0012	88	-0.11331
88	183.13	192.13	0.0012	80	0.14849
89	183.1944	192.1944	0.0013	80	0.49855
89	183.1944	192.1944	0.0013	83	0.23499
89	183.1944	192.1944	0.0013	84	-0.1803
89	183.1944	192.1944	0.0013	80	0.31402
89	183.1944	192.1944	0.0013	83	-0.17669
89	183.1944	192.1944	0.0013	84	0.13546
90	183.1945	192.1945	0.0371	80	-0.31421
90	183.1945	192.1945	0.0371	83	-0.17634
90	183.1945	192.1945	0.0371	84	0.1353
90	183.1945	192.1945	0.0371	80	0.49823
90	183.1945	192.1945	0.0371	83	-0.23542
90	183.1945	192.1945	0.0371	84	0.18048
91	183.2245	192.2245	0.0271	81	0.26513
91	183.2245	192.2245	0.0271	82	-0.26688
91	183.2245	192.2245	0.0271	85	0.13468
91	183.2245	192.2245	0.0271	87	0.17701
91	183.2245	192.2245	0.0271	81	0.14891
91	183.2245	192.2245	0.0271	82	0.42701
91	183.2245	192.2245	0.0271	85	-0.2135
92	183.2247	192.2247	0.0001	81	-0.1431
92	183.2247	192.2247	0.0001	82	0.42023
92	183.2247	192.2247	0.0001	85	-0.21053
92	183.2247	192.2247	0.0001	81	0.26199
92	183.2247	192.2247	0.0001	82	0.26767
92	183.2247	192.2247	0.0001	85	-0.13287
92	183.2247	192.2247	0.0001	87	0.17356
93	183.2251	192.2251	0.0038	81	0.14153
93	183.2251	192.2251	0.0038	82	-0.19061
93	183.2251	192.2251	0.0038	81	0.26675
93	183.2251	192.2251	0.0038	82	0.46539
93	183.2251	192.2251	0.0038	85	-0.23538
93	183.2251	192.2251	0.0038	87	0.17968
93	183.2251	192.2251	0.0038	83	-0.11257

94	183.2253	192.2253	0.0008	81	-0.26146
94	183.2253	192.2253	0.0008	82	0.45915
94	183.2253	192.2253	0.0008	85	-0.23373
94	183.2253	192.2253	0.0008	87	-0.17715
94	183.2253	192.2253	0.0008	81	0.13731
94	183.2253	192.2253	0.0008	82	0.18811
94	183.2253	192.2253	0.0008	82	0.10389
95	183.2526	192.2526	0.0023	81	0.10399
95	183.2526	192.2526	0.0023	85	-0.1696
95	183.2526	192.2526	0.0023	86	0.38092
95	183.2526	192.2526	0.0023	89	0.25714
95	183.2526	192.2526	0.0023	91	0.26034
95	183.2526	192.2526	0.0023	86	-0.2848
95	183.2526	192.2526	0.0023	89	-0.19181
95	183.2526	192.2526	0.0023	91	-0.12039
96	183.2531	192.2531	0.0032	86	0.2839
96	183.2531	192.2531	0.0032	89	0.18966
96	183.2531	192.2531	0.0032	91	-0.12101
96	183.2531	192.2531	0.0032	81	0.10322
96	183.2531	192.2531	0.0032	85	0.16883
96	183.2531	192.2531	0.0032	86	0.37988
96	183.2531	192.2531	0.0032	89	0.25319
96	183.2531	192.2531	0.0032	91	-0.26093
97	183.2547	192.2547	0.0026	80	-0.28422
97	183.2547	192.2547	0.0026	83	-0.17754
97	183.2547	192.2547	0.0026	84	0.58301
97	183.2547	192.2547	0.0026	88	-0.16127
97	183.2547	192.2547	0.0026	90	0.10491
98	183.2553	192.2553	0.0025	80	0.28509
98	183.2553	192.2553	0.0025	83	-0.17852
98	183.2553	192.2553	0.0025	84	0.58592
98	183.2553	192.2553	0.0025	88	-0.16081
98	183.2553	192.2553	0.0025	90	-0.10655
99	183.26	192.26	0.0033	81	0.11558
99	183.26	192.26	0.0033	85	-0.17257
99	183.26	192.26	0.0033	86	0.42949
99	183.26	192.26	0.0033	87	-0.10759
99	183.26	192.26	0.0033	89	0.28703
99	183.26	192.26	0.0033	91	0.26736
99	183.26	192.26	0.0033	86	-0.21281
99	183.26	192.26	0.0033	89	-0.14204
99	183.26	192.26	0.0033	91	-0.1036
100	183.2605	192.2605	0.004	86	0.21308
100	183.2605	192.2605	0.004	89	0.14089

100	183.2605	192.2605	0.004	91	-0.10448
100	183.2605	192.2605	0.004	81	0.11526
100	183.2605	192.2605	0.004	85	0.17262
100	183.2605	192.2605	0.004	86	0.43026
100	183.2605	192.2605	0.004	87	-0.10756
100	183.2605	192.2605	0.004	89	0.28412
100	183.2605	192.2605	0.004	91	-0.26927
101	183.3088	192.3088	0.0019	82	-0.17344
101	183.3088	192.3088	0.0019	80	0.1345
101	183.3088	192.3088	0.0019	84	0.62729
101	183.3088	192.3088	0.0019	88	-0.16009
102	183.3092	192.3092	0.0007	82	-0.23489
102	183.3092	192.3092	0.0007	80	-0.13048
102	183.3092	192.3092	0.0007	84	0.60457
102	183.3092	192.3092	0.0007	88	-0.15516
103	183.311	192.311	0.0005	83	0.12184
103	183.311	192.311	0.0005	83	0.11583
103	183.311	192.311	0.0005	82	-0.12596
103	183.311	192.311	0.0005	82	0.6305
104	183.3115	192.3115	0.0028	83	0.11672
104	183.3115	192.3115	0.0028	83	-0.10805
104	183.3115	192.3115	0.0028	82	0.62312
104	183.3115	192.3115	0.0028	82	0.12937
105	183.3121	192.3121	0.0004	82	0.59413
105	183.3121	192.3121	0.0004	82	-0.10222
105	183.3121	192.3121	0.0004	83	-0.13001
105	183.3121	192.3121	0.0004	84	0.22003
106	183.3125	192.3125	0.0019	82	0.61491
106	183.3125	192.3125	0.0019	83	0.14879
106	183.3125	192.3125	0.0019	84	0.10284
106	183.3125	192.3125	0.0019	84	0.16519
107	183.3182	192.3182	0.0231	81	-0.14927
107	183.3182	192.3182	0.0231	85	-0.20228
107	183.3182	192.3182	0.0231	81	0.18041
107	183.3182	192.3182	0.0231	87	-0.1089
107	183.3182	192.3182	0.0231	81	0.29303
107	183.3182	192.3182	0.0231	82	0.10528
107	183.3182	192.3182	0.0231	85	0.44073
107	183.3182	192.3182	0.0231	87	-0.17745
108	183.3184	192.3184	0.0071	81	0.14384
108	183.3184	192.3184	0.0071	85	-0.18375
108	183.3184	192.3184	0.0071	81	-0.29411
108	183.3184	192.3184	0.0071	82	0.10911
108	183.3184	192.3184	0.0071	85	0.45103

108	183.3184	192.3184	0.0071	87	0.1771
108	183.3184	192.3184	0.0071	81	0.18764
108	183.3184	192.3184	0.0071	87	-0.11339
109	183.3187	192.3187	0.0164	81	0.33393
109	183.3187	192.3187	0.0164	82	0.10708
109	183.3187	192.3187	0.0164	85	0.43546
109	183.3187	192.3187	0.0164	87	-0.20088
109	183.3187	192.3187	0.0164	89	-0.11121
109	183.3187	192.3187	0.0164	81	0.13345
109	183.3187	192.3187	0.0164	85	0.20966
110	183.3189	192.3189	0.0082	81	-0.33637
110	183.3189	192.3189	0.0082	82	0.11061
110	183.3189	192.3189	0.0082	85	0.44241
110	183.3189	192.3189	0.0082	87	0.20132
110	183.3189	192.3189	0.0082	89	0.11167
110	183.3189	192.3189	0.0082	81	-0.13304
110	183.3189	192.3189	0.0082	85	0.18641
111	183.4078	192.4078	0.0102	81	0.15413
111	183.4078	192.4078	0.0102	82	-0.15774
111	183.4078	192.4078	0.0102	85	-0.1411
111	183.4078	192.4078	0.0102	86	0.43209
111	183.4078	192.4078	0.0102	87	-0.13476
111	183.4078	192.4078	0.0102	89	0.35013
111	183.4078	192.4078	0.0102	91	0.29322
112	183.4081	192.4081	0.0145	81	0.15369
112	183.4081	192.4081	0.0145	82	0.15764
112	183.4081	192.4081	0.0145	85	0.14098
112	183.4081	192.4081	0.0145	86	0.43268
112	183.4081	192.4081	0.0145	87	-0.13497
112	183.4081	192.4081	0.0145	89	0.34731
112	183.4081	192.4081	0.0145	91	-0.2959
113	183.433	192.433	0.0256	81	0.11842
113	183.433	192.433	0.0256	85	0.18316
113	183.433	192.433	0.0256	91	-0.11205
113	183.433	192.433	0.0256	81	0.62378
113	183.433	192.433	0.0256	89	-0.13616
114	183.4337	192.4337	0	81	-0.1249
114	183.4337	192.4337	0	81	0.61275
114	183.4337	192.4337	0	89	-0.13388
114	183.4337	192.4337	0	81	-0.11574
114	183.4337	192.4337	0	85	0.18355
114	183.4337	192.4337	0	91	-0.11344
115	183.4339	192.4339	0	81	0.62769
115	183.4339	192.4339	0	85	0.11956

115	183.4339	192.4339	0	89	-0.13864
115	183.4339	192.4339	0	85	-0.14556
116	183.434	192.434	0.0001	85	-0.14499
116	183.434	192.434	0.0001	81	0.61801
116	183.434	192.434	0.0001	85	-0.11884
116	183.434	192.434	0.0001	89	-0.13539
116	183.434	192.434	0.0001	81	0.12485
117	183.4475	192.4475	0.0033	82	-0.10687
117	183.4475	192.4475	0.0033	86	-0.17686
117	183.4475	192.4475	0.0033	87	-0.1892
117	183.4475	192.4475	0.0033	89	0.1415
117	183.4475	192.4475	0.0033	91	-0.1392
117	183.4475	192.4475	0.0033	88	0.20738
117	183.4475	192.4475	0.0033	90	-0.15118
117	183.4475	192.4475	0.0033	83	0.10696
117	183.4475	192.4475	0.0033	84	0.16157
117	183.4475	192.4475	0.0033	88	0.40043
117	183.4475	192.4475	0.0033	90	-0.2217
118	183.4483	192.4483	0.0043	82	0.11229
118	183.4483	192.4483	0.0043	86	-0.1866
118	183.4483	192.4483	0.0043	87	-0.19713
118	183.4483	192.4483	0.0043	89	0.1489
118	183.4483	192.4483	0.0043	91	0.14547
118	183.4483	192.4483	0.0043	83	0.10756
118	183.4483	192.4483	0.0043	84	0.16282
118	183.4483	192.4483	0.0043	88	0.40294
118	183.4483	192.4483	0.0043	90	0.22948
118	183.4483	192.4483	0.0043	88	-0.21365
118	183.4483	192.4483	0.0043	90	-0.15284
119	183.4487	192.4487	0.0003	88	0.14779
119	183.4487	192.4487	0.0003	90	-0.11063
119	183.4487	192.4487	0.0003	83	0.10111
119	183.4487	192.4487	0.0003	84	0.15715
119	183.4487	192.4487	0.0003	88	0.38989
119	183.4487	192.4487	0.0003	90	-0.2188
119	183.4487	192.4487	0.0003	86	-0.10597
119	183.4487	192.4487	0.0003	87	-0.10742
119	183.4487	192.4487	0.0003	82	-0.10933
119	183.4487	192.4487	0.0003	86	-0.20259
119	183.4487	192.4487	0.0003	87	-0.2051
119	183.4487	192.4487	0.0003	89	0.15488
119	183.4487	192.4487	0.0003	91	-0.14836
120	183.4492	192.4492	0.0001	83	0.10196
120	183.4492	192.4492	0.0001	84	0.15904

120	183.4492	192.4492	0.0001	88	0.39435
120	183.4492	192.4492	0.0001	90	0.22424
120	183.4492	192.4492	0.0001	88	-0.14774
120	183.4492	192.4492	0.0001	90	-0.1141
120	183.4492	192.4492	0.0001	82	0.11433
120	183.4492	192.4492	0.0001	86	-0.21429
120	183.4492	192.4492	0.0001	87	-0.21447
120	183.4492	192.4492	0.0001	89	0.16297
120	183.4492	192.4492	0.0001	91	0.15385
120	183.4492	192.4492	0.0001	86	0.11266
120	183.4492	192.4492	0.0001	87	0.11263
120	183.4492	192.4492	0.0001	91	-0.10361
121	183.4947	192.4947	0.0038	90	-0.1168
121	183.4947	192.4947	0.0038	83	0.11407
121	183.4947	192.4947	0.0038	84	0.13974
121	183.4947	192.4947	0.0038	88	0.31931
121	183.4947	192.4947	0.0038	90	-0.19271
121	183.4947	192.4947	0.0038	86	0.14575
121	183.4947	192.4947	0.0038	87	0.1054
121	183.4947	192.4947	0.0038	91	0.11979
121	183.4947	192.4947	0.0038	86	0.33316
121	183.4947	192.4947	0.0038	87	0.24041
121	183.4947	192.4947	0.0038	89	-0.18794
121	183.4947	192.4947	0.0038	91	0.15395
122	183.4951	192.4951	0.0063	83	0.11503
122	183.4951	192.4951	0.0063	84	0.14139
122	183.4951	192.4951	0.0063	88	0.32262
122	183.4951	192.4951	0.0063	90	0.19833
122	183.4951	192.4951	0.0063	90	-0.11842
122	183.4951	192.4951	0.0063	86	0.33093
122	183.4951	192.4951	0.0063	87	0.23753
122	183.4951	192.4951	0.0063	89	-0.18713
122	183.4951	192.4951	0.0063	91	-0.14988
122	183.4951	192.4951	0.0063	86	-0.14385
122	183.4951	192.4951	0.0063	87	-0.10303
122	183.4951	192.4951	0.0063	91	0.11815
123	183.4984	192.4984	0.0008	86	0.11108
123	183.4984	192.4984	0.0008	91	0.1102
123	183.4984	192.4984	0.0008	82	0.10084
123	183.4984	192.4984	0.0008	86	0.36892
123	183.4984	192.4984	0.0008	87	0.27735
123	183.4984	192.4984	0.0008	89	-0.21538
123	183.4984	192.4984	0.0008	91	0.18563
123	183.4984	192.4984	0.0008	88	0.11397

123	183.4984	192.4984	0.0008	90	-0.12115
123	183.4984	192.4984	0.0008	84	0.11354
123	183.4984	192.4984	0.0008	88	0.25979
123	183.4984	192.4984	0.0008	90	-0.15497
124	183.4986	192.4986	0.0015	86	0.36724
124	183.4986	192.4986	0.0015	87	0.27465
124	183.4986	192.4986	0.0015	89	-0.21515
124	183.4986	192.4986	0.0015	91	-0.18182
124	183.4986	192.4986	0.0015	86	-0.11014
124	183.4986	192.4986	0.0015	91	0.10902
124	183.4986	192.4986	0.0015	84	0.11509
124	183.4986	192.4986	0.0015	88	0.26297
124	183.4986	192.4986	0.0015	90	0.15974
124	183.4986	192.4986	0.0015	88	-0.11623
124	183.4986	192.4986	0.0015	90	-0.12349
125	183.5207	192.5207	0.0072	81	-0.12473
125	183.5207	192.5207	0.0072	83	0.15337
125	183.5207	192.5207	0.0072	81	0.21241
125	183.5207	192.5207	0.0072	81	0.37147
125	183.5207	192.5207	0.0072	82	-0.30092
125	183.5207	192.5207	0.0072	83	-0.16248
125	183.5207	192.5207	0.0072	85	-0.18643
125	183.5207	192.5207	0.0072	86	-0.14008
125	183.5207	192.5207	0.0072	87	0.10112
125	183.5207	192.5207	0.0072	91	-0.10935
126	183.5209	192.5209	0.0024	81	-0.13731
126	183.5209	192.5209	0.0024	83	-0.14138
126	183.5209	192.5209	0.0024	81	0.36533
126	183.5209	192.5209	0.0024	82	0.30004
126	183.5209	192.5209	0.0024	83	0.17354
126	183.5209	192.5209	0.0024	85	0.1863
126	183.5209	192.5209	0.0024	86	-0.1374
126	183.5209	192.5209	0.0024	91	0.10957
126	183.5209	192.5209	0.0024	81	-0.21526
127	183.5213	192.5213	0.0032	81	0.10172
127	183.5213	192.5213	0.0032	81	0.41209
127	183.5213	192.5213	0.0032	82	-0.29409
127	183.5213	192.5213	0.0032	83	0.15701
127	183.5213	192.5213	0.0032	85	-0.18335
127	183.5213	192.5213	0.0032	86	-0.15519
127	183.5213	192.5213	0.0032	87	0.11215
127	183.5213	192.5213	0.0032	91	-0.10759
127	183.5213	192.5213	0.0032	81	0.11721
127	183.5213	192.5213	0.0032	83	0.15887

128	183.5216	192.5216	0.0023	81	0.40563
128	183.5216	192.5216	0.0023	82	0.29095
128	183.5216	192.5216	0.0023	83	-0.16938
128	183.5216	192.5216	0.0023	85	0.18139
128	183.5216	192.5216	0.0023	86	-0.15234
128	183.5216	192.5216	0.0023	87	0.11004
128	183.5216	192.5216	0.0023	91	0.10688
128	183.5216	192.5216	0.0023	81	-0.10538
128	183.5216	192.5216	0.0023	81	0.13144
128	183.5216	192.5216	0.0023	83	-0.14728
129	183.5335	192.5335	0.011	80	-0.13278
129	183.5335	192.5335	0.011	80	-0.13545
129	183.5335	192.5335	0.011	83	-0.21789
129	183.5335	192.5335	0.011	85	0.42697
129	183.5335	192.5335	0.011	81	-0.22057
129	183.5335	192.5335	0.011	85	-0.28155
129	183.5335	192.5335	0.011	86	0.11068
129	183.5335	192.5335	0.011	87	-0.19319
130	183.534	192.534	0.0035	81	0.13932
130	183.534	192.534	0.0035	87	0.11783
130	183.534	192.534	0.0035	81	0.18097
130	183.534	192.534	0.0035	85	0.5012
130	183.534	192.534	0.0035	87	0.15435
130	183.534	192.534	0.0035	83	-0.16423
130	183.534	192.534	0.0035	80	0.17801
130	183.534	192.534	0.0035	83	0.14429
130	183.534	192.534	0.0035	81	-0.1114
131	183.5344	192.5344	0.002	80	-0.13938
131	183.5344	192.5344	0.002	83	0.22787
131	183.5344	192.5344	0.002	80	0.13574
131	183.5344	192.5344	0.002	81	-0.21585
131	183.5344	192.5344	0.002	85	0.2798
131	183.5344	192.5344	0.002	86	0.10901
131	183.5344	192.5344	0.002	87	-0.19092
131	183.5344	192.5344	0.002	85	0.43041
132	183.5347	192.5347	0.0095	81	-0.18437
132	183.5347	192.5347	0.0095	85	0.50113
132	183.5347	192.5347	0.0095	87	-0.15777
132	183.5347	192.5347	0.0095	81	0.13327
132	183.5347	192.5347	0.0095	87	0.11496
132	183.5347	192.5347	0.0095	80	-0.18214
132	183.5347	192.5347	0.0095	83	0.14826
132	183.5347	192.5347	0.0095	83	0.17578
132	183.5347	192.5347	0.0095	81	0.10867

133	183.5979	192.5979	0.0004	81	0.10743
133	183.5979	192.5979	0.0004	82	0.20768
133	183.5979	192.5979	0.0004	80	0.20742
133	183.5979	192.5979	0.0004	82	-0.15105
133	183.5979	192.5979	0.0004	83	0.51543
133	183.5979	192.5979	0.0004	88	-0.15062
133	183.5979	192.5979	0.0004	90	0.15465
133	183.5979	192.5979	0.0004	83	-0.18543
134	183.5983	192.5983	0.0005	81	-0.108
134	183.5983	192.5983	0.0005	82	0.20995
134	183.5983	192.5983	0.0005	83	0.18589
134	183.5983	192.5983	0.0005	80	-0.20605
134	183.5983	192.5983	0.0005	82	-0.14406
134	183.5983	192.5983	0.0005	83	0.51453
134	183.5983	192.5983	0.0005	88	-0.14832
134	183.5983	192.5983	0.0005	90	-0.15472
135	183.5989	192.5989	0.0007	80	0.20983
135	183.5989	192.5989	0.0007	82	0.15859
135	183.5989	192.5989	0.0007	83	0.54704
135	183.5989	192.5989	0.0007	88	-0.15963
135	183.5989	192.5989	0.0007	90	0.15656
135	183.5989	192.5989	0.0007	81	0.1007
135	183.5989	192.5989	0.0007	82	0.1949
136	183.5993	192.5993	0.0006	80	-0.20876
136	183.5993	192.5993	0.0006	82	0.1538
136	183.5993	192.5993	0.0006	83	0.54607
136	183.5993	192.5993	0.0006	88	-0.15725
136	183.5993	192.5993	0.0006	90	-0.15687
136	183.5993	192.5993	0.0006	81	-0.10149
136	183.5993	192.5993	0.0006	82	0.19938
137	183.621	192.621	0.0001	85	-0.13473
137	183.621	192.621	0.0001	82	-0.11374
137	183.621	192.621	0.0001	85	-0.1973
137	183.621	192.621	0.0001	80	-0.14279
137	183.621	192.621	0.0001	83	-0.2276
137	183.621	192.621	0.0001	80	0.13587
137	183.621	192.621	0.0001	83	0.49401
137	183.621	192.621	0.0001	84	-0.1115
138	183.6218	192.6218	0.0002	82	-0.11702
138	183.6218	192.6218	0.0002	85	-0.20327
138	183.6218	192.6218	0.0002	83	0.21104
138	183.6218	192.6218	0.0002	80	-0.13229
138	183.6218	192.6218	0.0002	83	0.45193
138	183.6218	192.6218	0.0002	84	-0.10264

138	183.6218	192.6218	0.0002	85	0.13874
138	183.6218	192.6218	0.0002	80	-0.1159
138	183.6218	192.6218	0.0002	83	0.24274
139	183.622	192.622	0.0003	83	0.20885
139	183.622	192.622	0.0003	80	0.16956
139	183.622	192.622	0.0003	83	0.47052
139	183.622	192.622	0.0003	84	-0.10558
139	183.622	192.622	0.0003	85	-0.14376
139	183.622	192.622	0.0003	83	-0.21051
139	183.622	192.622	0.0003	82	-0.10988
139	183.622	192.622	0.0003	85	-0.18952
140	183.6227	192.6227	0.0004	80	-0.17897
140	183.6227	192.6227	0.0004	83	0.51193
140	183.6227	192.6227	0.0004	84	-0.11542
140	183.6227	192.6227	0.0004	83	-0.18772
140	183.6227	192.6227	0.0004	82	-0.10754
140	183.6227	192.6227	0.0004	85	-0.18508
140	183.6227	192.6227	0.0004	85	-0.14774
141	183.6231	192.6231	0.0063	83	0.23927
141	183.6231	192.6231	0.0063	84	0.26865
141	183.6231	192.6231	0.0063	88	0.50654
141	183.6231	192.6231	0.0063	90	-0.32192
142	183.6242	192.6242	0.0068	83	0.23899
142	183.6242	192.6242	0.0068	84	0.26817
142	183.6242	192.6242	0.0068	88	0.50449
142	183.6242	192.6242	0.0068	90	0.32621
143	183.664	192.664	0.001	85	-0.19803
143	183.664	192.664	0.001	86	0.49246
143	183.664	192.664	0.001	87	-0.21465
143	183.664	192.664	0.001	89	0.27276
143	183.664	192.664	0.001	91	0.25657
144	183.6653	192.6653	0.0007	85	0.19832
144	183.6653	192.6653	0.0007	86	0.4932
144	183.6653	192.6653	0.0007	87	-0.21471
144	183.6653	192.6653	0.0007	89	0.26975
144	183.6653	192.6653	0.0007	91	-0.25798
145	183.6899	192.6899	0.0001	85	0.26573
145	183.6899	192.6899	0.0001	87	0.40543
145	183.6899	192.6899	0.0001	89	0.28724
145	183.6899	192.6899	0.0001	91	0.15274
145	183.6899	192.6899	0.0001	85	-0.13515
145	183.6899	192.6899	0.0001	87	-0.28036
145	183.6899	192.6899	0.0001	89	-0.19864
146	183.6905	192.6905	0.0001	85	-0.13494

146	183.6905	192.6905	0.0001	87	0.28029
146	183.6905	192.6905	0.0001	89	0.19829
146	183.6905	192.6905	0.0001	85	-0.26531
146	183.6905	192.6905	0.0001	87	0.40518
146	183.6905	192.6905	0.0001	89	0.28665
146	183.6905	192.6905	0.0001	91	-0.15525
147	183.6958	192.6958	0.0004	85	0.27685
147	183.6958	192.6958	0.0004	86	-0.10501
147	183.6958	192.6958	0.0004	87	0.4428
147	183.6958	192.6958	0.0004	89	0.31447
147	183.6958	192.6958	0.0004	91	0.15955
147	183.6958	192.6958	0.0004	85	-0.10991
147	183.6958	192.6958	0.0004	87	-0.21491
147	183.6958	192.6958	0.0004	89	-0.15265
148	183.6964	192.6964	0.0005	85	-0.10972
148	183.6964	192.6964	0.0005	87	0.21487
148	183.6964	192.6964	0.0005	89	0.15237
148	183.6964	192.6964	0.0005	85	-0.27641
148	183.6964	192.6964	0.0005	86	-0.1049
148	183.6964	192.6964	0.0005	87	0.44251
148	183.6964	192.6964	0.0005	89	0.31385
148	183.6964	192.6964	0.0005	91	-0.16228
149	183.7097	192.7097	0.0035	82	0.14164
149	183.7097	192.7097	0.0035	86	0.46813
149	183.7097	192.7097	0.0035	87	0.33844
149	183.7097	192.7097	0.0035	89	-0.25797
149	183.7097	192.7097	0.0035	91	0.24808
150	183.7098	192.7098	0.0043	82	-0.14174
150	183.7098	192.7098	0.0043	86	0.4686
150	183.7098	192.7098	0.0043	87	0.33798
150	183.7098	192.7098	0.0043	89	-0.25987
150	183.7098	192.7098	0.0043	91	-0.24583
151	183.7656	192.7656	0.0009	82	-0.10191
151	183.7656	192.7656	0.0009	80	0.13124
151	183.7656	192.7656	0.0009	84	-0.13987
151	183.7656	192.7656	0.0009	83	0.15793
151	183.7656	192.7656	0.0009	84	0.5902
151	183.7656	192.7656	0.0009	88	-0.15526
152	183.7664	192.7664	0.0004	82	-0.10433
152	183.7664	192.7664	0.0004	83	0.15758
152	183.7664	192.7664	0.0004	84	0.58666
152	183.7664	192.7664	0.0004	88	-0.15376
152	183.7664	192.7664	0.0004	80	0.1284
152	183.7664	192.7664	0.0004	84	0.13999

153	183.7666	192.7666	0.0023	84	0.15191
153	183.7666	192.7666	0.0023	80	-0.13314
153	183.7666	192.7666	0.0023	83	0.15598
153	183.7666	192.7666	0.0023	84	0.57842
153	183.7666	192.7666	0.0023	88	-0.15245
153	183.7666	192.7666	0.0023	82	-0.10395
153	183.7666	192.7666	0.0023	84	0.11027
154	183.7672	192.7672	0.0015	80	0.13562
154	183.7672	192.7672	0.0015	83	0.15691
154	183.7672	192.7672	0.0015	84	0.58061
154	183.7672	192.7672	0.0015	88	-0.15197
154	183.7672	192.7672	0.0015	84	-0.15323
154	183.7672	192.7672	0.0015	82	-0.10528
154	183.7672	192.7672	0.0015	84	0.11882
155	183.7811	192.7811	0.0007	80	-0.27652
155	183.7811	192.7811	0.0007	84	0.44909
155	183.7811	192.7811	0.0007	88	-0.11864
155	183.7811	192.7811	0.0007	90	0.10694
155	183.7811	192.7811	0.0007	84	-0.36334
156	183.7813	192.7813	0.0024	84	0.36303
156	183.7813	192.7813	0.0024	80	0.27626
156	183.7813	192.7813	0.0024	84	0.44917
156	183.7813	192.7813	0.0024	88	-0.11727
156	183.7813	192.7813	0.0024	90	-0.1078
157	183.7827	192.7827	0.0057	81	-0.15895
157	183.7827	192.7827	0.0057	82	0.15104
157	183.7827	192.7827	0.0057	84	-0.10794
157	183.7827	192.7827	0.0057	80	-0.26461
157	183.7827	192.7827	0.0057	84	0.53695
157	183.7827	192.7827	0.0057	88	-0.14203
157	183.7827	192.7827	0.0057	90	0.10231
158	183.7828	192.7828	0.0034	81	0.10661
158	183.7828	192.7828	0.0034	82	0.10298
158	183.7828	192.7828	0.0034	84	-0.1181
158	183.7828	192.7828	0.0034	80	0.27148
158	183.7828	192.7828	0.0034	83	-0.10164
158	183.7828	192.7828	0.0034	84	0.55265
158	183.7828	192.7828	0.0034	88	-0.14478
158	183.7828	192.7828	0.0034	90	-0.10605
159	183.7839	192.7839	0.0009	81	0.48185
159	183.7839	192.7839	0.0009	82	-0.44935
159	183.7839	192.7839	0.0009	84	0.18258
160	183.7845	192.7845	0.0013	81	0.49696
160	183.7845	192.7845	0.0013	82	0.46283

160	183.7845	192.7845	0.0013	84	-0.12249
161	183.8271	192.8271	0.0058	83	0.14819
161	183.8271	192.8271	0.0058	84	0.17234
161	183.8271	192.8271	0.0058	88	0.51445
161	183.8271	192.8271	0.0058	90	-0.35961
161	183.8271	192.8271	0.0058	88	0.18755
162	183.8275	192.8275	0.004	88	-0.18672
162	183.8275	192.8275	0.004	83	0.14774
162	183.8275	192.8275	0.004	84	0.17199
162	183.8275	192.8275	0.004	88	0.51201
162	183.8275	192.8275	0.004	90	0.36364
163	183.8409	192.8409	0.003	91	0.1029
163	183.8409	192.8409	0.003	82	-0.14208
163	183.8409	192.8409	0.003	86	0.44714
163	183.8409	192.8409	0.003	87	0.40067
163	183.8409	192.8409	0.003	89	-0.19922
163	183.8409	192.8409	0.003	91	-0.2269
164	183.8419	192.8419	0.0015	82	0.14195
164	183.8419	192.8419	0.0015	86	0.4464
164	183.8419	192.8419	0.0015	87	0.40129
164	183.8419	192.8419	0.0015	89	-0.19735
164	183.8419	192.8419	0.0015	91	0.22877
164	183.8419	192.8419	0.0015	91	0.10338
165	183.8904	192.8904	0.0242	81	-0.17024
165	183.8904	192.8904	0.0242	82	-0.21489
165	183.8904	192.8904	0.0242	85	0.53556
165	183.8904	192.8904	0.0242	87	0.30854
165	183.8904	192.8904	0.0242	89	0.13426
166	183.8912	192.8912	0.0186	81	0.16993
166	183.8912	192.8912	0.0186	82	-0.21585
166	183.8912	192.8912	0.0186	85	0.53493
166	183.8912	192.8912	0.0186	87	-0.30892
166	183.8912	192.8912	0.0186	89	-0.13472
167	183.9094	192.9094	0.0009	85	0.33749
167	183.9094	192.9094	0.0009	86	-0.12611
167	183.9094	192.9094	0.0009	87	0.49024
167	183.9094	192.9094	0.0009	89	0.31874
167	183.9094	192.9094	0.0009	91	0.14884
168	183.9097	192.9097	0.001	85	-0.33715
168	183.9097	192.9097	0.001	86	-0.12612
168	183.9097	192.9097	0.001	87	0.49017
168	183.9097	192.9097	0.001	89	0.31817
168	183.9097	192.9097	0.001	91	-0.15148
169	183.9805	192.9805	0.0001	88	-0.32555

169	183.9805	192.9805	0.0001	90	0.49926
169	183.9805	192.9805	0.0001	88	0.18581
169	183.9805	192.9805	0.0001	90	-0.31098
170	183.982	192.982	0.0002	88	0.18336
170	183.982	192.982	0.0002	90	0.31113
170	183.982	192.982	0.0002	88	0.32053
170	183.982	192.982	0.0002	90	0.5008
171	183.9853	192.9853	0.0028	81	0.12434
171	183.9853	192.9853	0.0028	86	0.26364
171	183.9853	192.9853	0.0028	87	-0.12911
171	183.9853	192.9853	0.0028	89	0.22182
171	183.9853	192.9853	0.0028	91	0.28927
171	183.9853	192.9853	0.0028	86	-0.21115
171	183.9853	192.9853	0.0028	87	0.10346
171	183.9853	192.9853	0.0028	89	-0.17674
171	183.9853	192.9853	0.0028	86	0.14666
171	183.9853	192.9853	0.0028	89	0.12303
171	183.9853	192.9853	0.0028	91	0.12685
171	183.9853	192.9853	0.0028	88	0.10809
171	183.9853	192.9853	0.0028	90	-0.15434
172	183.9855	192.9855	0.0097	86	-0.13573
172	183.9855	192.9855	0.0097	89	-0.11364
172	183.9855	192.9855	0.0097	91	-0.13433
172	183.9855	192.9855	0.0097	81	0.1567
172	183.9855	192.9855	0.0097	86	0.33435
172	183.9855	192.9855	0.0097	87	-0.16361
172	183.9855	192.9855	0.0097	89	0.27932
172	183.9855	192.9855	0.0097	91	0.19479
172	183.9855	192.9855	0.0097	86	0.10931
172	183.9855	192.9855	0.0097	91	0.23697
172	183.9855	192.9855	0.0097	90	0.11105
173	183.986	192.986	0.0164	86	-0.16444
173	183.986	192.986	0.0164	89	-0.13706
173	183.986	192.986	0.0164	81	-0.1297
173	183.986	192.986	0.0164	86	-0.27634
173	183.986	192.986	0.0164	87	0.13532
173	183.986	192.986	0.0164	89	-0.22932
173	183.986	192.986	0.0164	91	0.28984
173	183.986	192.986	0.0164	91	-0.12933
173	183.986	192.986	0.0164	86	-0.19843
173	183.986	192.986	0.0164	89	-0.16485
173	183.986	192.986	0.0164	90	0.13786
174	183.9863	192.9863	0.0211	86	-0.1386
174	183.9863	192.9863	0.0211	89	-0.11489

174	183.9863	192.9863	0.0211	86	-0.12099
174	183.9863	192.9863	0.0211	91	0.19323
174	183.9863	192.9863	0.0211	81	0.12408
174	183.9863	192.9863	0.0211	86	0.26689
174	183.9863	192.9863	0.0211	87	-0.13027
174	183.9863	192.9863	0.0211	89	0.22074
174	183.9863	192.9863	0.0211	91	-0.17188
174	183.9863	192.9863	0.0211	88	-0.17272
174	183.9863	192.9863	0.0211	90	0.3189
174	183.9863	192.9863	0.0211	88	0.11708
175	183.9864	192.9864	0.0068	86	0.19128
175	183.9864	192.9864	0.0068	89	0.1597
175	183.9864	192.9864	0.0068	91	-0.1535
175	183.9864	192.9864	0.0068	91	-0.13273
175	183.9864	192.9864	0.0068	86	-0.15854
175	183.9864	192.9864	0.0068	89	-0.13097
175	183.9864	192.9864	0.0068	88	-0.26955
175	183.9864	192.9864	0.0068	90	0.36669
175	183.9864	192.9864	0.0068	90	-0.23806
176	183.9878	192.9878	0.0001	88	0.14103
176	183.9878	192.9878	0.0001	90	0.24129
176	183.9878	192.9878	0.0001	88	0.33406
176	183.9878	192.9878	0.0001	90	0.52582
177	183.9925	192.9925	0.0033	85	0.22693
177	183.9925	192.9925	0.0033	87	0.41569
177	183.9925	192.9925	0.0033	89	0.41494
177	183.9925	192.9925	0.0033	91	0.23896
178	183.9941	192.9941	0.0026	85	-0.22651
178	183.9941	192.9941	0.0026	87	0.41679
178	183.9941	192.9941	0.0026	89	0.41511
178	183.9941	192.9941	0.0026	91	-0.24295
179	184.0267	193.0267	0.006	84	0.12344
179	184.0267	193.0267	0.006	81	0.12293
179	184.0267	193.0267	0.006	82	-0.13211
179	184.0267	193.0267	0.006	85	-0.12198
179	184.0267	193.0267	0.006	86	0.46295
179	184.0267	193.0267	0.006	87	-0.12887
179	184.0267	193.0267	0.006	89	0.30046
179	184.0267	193.0267	0.006	91	0.2474
180	184.0276	193.0276	0.0053	84	-0.11688
180	184.0276	193.0276	0.0053	86	-0.11614
180	184.0276	193.0276	0.0053	81	0.11796
180	184.0276	193.0276	0.0053	82	0.12849
180	184.0276	193.0276	0.0053	85	0.11878

180	184.0276	193.0276	0.0053	86	0.44589
180	184.0276	193.0276	0.0053	87	-0.12409
180	184.0276	193.0276	0.0053	89	0.28658
180	184.0276	193.0276	0.0053	91	-0.24283
180	184.0276	193.0276	0.0053	86	-0.10518
181	184.0276	193.0276	0.0055	81	0.12114
181	184.0276	193.0276	0.0055	82	-0.13235
181	184.0276	193.0276	0.0055	85	-0.12131
181	184.0276	193.0276	0.0055	86	0.45529
181	184.0276	193.0276	0.0055	87	-0.12683
181	184.0276	193.0276	0.0055	89	0.29574
181	184.0276	193.0276	0.0055	91	0.24759
181	184.0276	193.0276	0.0055	86	0.1304
181	184.0276	193.0276	0.0055	84	0.10927
182	184.0285	193.0285	0.0035	81	0.12487
182	184.0285	193.0285	0.0035	82	0.13859
182	184.0285	193.0285	0.0035	85	0.12702
182	184.0285	193.0285	0.0035	86	0.47135
182	184.0285	193.0285	0.0035	87	-0.13122
182	184.0285	193.0285	0.0035	89	0.30314
182	184.0285	193.0285	0.0035	91	-0.26135
182	184.0285	193.0285	0.0035	84	-0.11626
183	184.117	193.117	0	89	0.22354
183	184.117	193.117	0	91	0.27276
183	184.117	193.117	0	87	-0.10227
183	184.117	193.117	0	89	0.36484
183	184.117	193.117	0	91	0.46991
184	184.1183	193.1183	0	87	0.10212
184	184.1183	193.1183	0	89	-0.36126
184	184.1183	193.1183	0	91	0.4729
184	184.1183	193.1183	0	89	0.22132
184	184.1183	193.1183	0	91	-0.27449
185	184.1227	193.1227	0	89	0.1751
185	184.1227	193.1227	0	91	0.21586
185	184.1227	193.1227	0	87	-0.10969
185	184.1227	193.1227	0	89	0.39069
185	184.1227	193.1227	0	91	0.49867
186	184.1239	193.1239	0	87	0.10952
186	184.1239	193.1239	0	89	-0.38685
186	184.1239	193.1239	0	91	0.50183
186	184.1239	193.1239	0	89	0.17337
186	184.1239	193.1239	0	91	-0.21722
187	184.1494	193.1494	0.0033	80	0.24888
187	184.1494	193.1494	0.0033	83	0.57273

187	184.1494	193.1494	0.0033	84	0.15145
187	184.1494	193.1494	0.0033	88	-0.20595
187	184.1494	193.1494	0.0033	90	0.19755
188	184.1511	193.1511	0.0035	80	-0.24867
188	184.1511	193.1511	0.0035	83	0.57335
188	184.1511	193.1511	0.0035	84	0.15122
188	184.1511	193.1511	0.0035	88	-0.20355
188	184.1511	193.1511	0.0035	90	-0.19869
189	184.1673	193.1673	0.0006	86	0.12858
189	184.1673	193.1673	0.0006	83	0.17438
189	184.1673	193.1673	0.0006	84	0.20644
189	184.1673	193.1673	0.0006	88	0.46313
189	184.1673	193.1673	0.0006	90	-0.33382
189	184.1673	193.1673	0.0006	88	-0.13564
190	184.1681	193.1681	0.0004	86	-0.13572
190	184.1681	193.1681	0.0004	88	0.13502
190	184.1681	193.1681	0.0004	83	0.1739
190	184.1681	193.1681	0.0004	84	0.20587
190	184.1681	193.1681	0.0004	88	0.46113
190	184.1681	193.1681	0.0004	90	0.3371
191	184.1686	193.1686	0.0033	83	0.18152
191	184.1686	193.1686	0.0033	84	0.21498
191	184.1686	193.1686	0.0033	88	0.48165
191	184.1686	193.1686	0.0033	90	-0.34065
191	184.1686	193.1686	0.0033	86	0.1249
192	184.1693	193.1693	0.0034	83	0.18097
192	184.1693	193.1693	0.0034	84	0.21433
192	184.1693	193.1693	0.0034	88	0.47971
192	184.1693	193.1693	0.0034	90	0.34478
192	184.1693	193.1693	0.0034	86	-0.132
193	184.2184	193.2184	0.0001	88	-0.38432
193	184.2184	193.2184	0.0001	90	0.58141
194	184.2196	193.2196	0.0001	88	0.37958
194	184.2196	193.2196	0.0001	90	0.58458
195	184.2276	193.2276	0.0002	88	0.11028
195	184.2276	193.2276	0.0002	90	-0.12908
195	184.2276	193.2276	0.0002	88	-0.37293
195	184.2276	193.2276	0.0002	90	0.57391
196	184.2301	193.2301	0.0002	88	0.36879
196	184.2301	193.2301	0.0002	90	0.57691
196	184.2301	193.2301	0.0002	88	0.1088
196	184.2301	193.2301	0.0002	90	0.1296
197	184.2339	193.2339	0.001	81	0.44512
197	184.2339	193.2339	0.001	82	0.44492

197	184.2339	193.2339	0.001	85	0.23402
197	184.2339	193.2339	0.001	86	-0.15608
197	184.2339	193.2339	0.001	87	0.12532
198	184.2346	193.2346	0.001	81	-0.44478
198	184.2346	193.2346	0.001	82	0.44478
198	184.2346	193.2346	0.001	85	0.23447
198	184.2346	193.2346	0.001	86	0.15633
198	184.2346	193.2346	0.001	87	-0.12576
199	184.2403	193.2403	0.0065	82	-0.10704
199	184.2403	193.2403	0.0065	86	-0.14475
199	184.2403	193.2403	0.0065	91	-0.11261
199	184.2403	193.2403	0.0065	88	-0.26896
199	184.2403	193.2403	0.0065	90	0.20329
199	184.2403	193.2403	0.0065	84	0.1328
199	184.2403	193.2403	0.0065	88	0.42353
199	184.2403	193.2403	0.0065	90	-0.2729
200	184.2416	193.2416	0.0005	88	0.12808
200	184.2416	193.2416	0.0005	90	-0.16551
200	184.2416	193.2416	0.0005	83	0.10211
200	184.2416	193.2416	0.0005	84	0.15127
200	184.2416	193.2416	0.0005	88	0.48223
200	184.2416	193.2416	0.0005	90	-0.29649
200	184.2416	193.2416	0.0005	86	-0.14056
201	184.2417	193.2417	0.0056	82	-0.108
201	184.2417	193.2417	0.0056	86	0.14971
201	184.2417	193.2417	0.0056	87	0.10036
201	184.2417	193.2417	0.0056	91	-0.1137
201	184.2417	193.2417	0.0056	84	0.13136
201	184.2417	193.2417	0.0056	88	0.41776
201	184.2417	193.2417	0.0056	90	0.2739
201	184.2417	193.2417	0.0056	88	0.27214
201	184.2417	193.2417	0.0056	90	0.20852
202	184.243	193.243	0.0003	83	0.10235
202	184.243	193.243	0.0003	84	0.15182
202	184.243	193.243	0.0003	88	0.48367
202	184.243	193.243	0.0003	90	0.30099
202	184.243	193.243	0.0003	88	-0.1231
202	184.243	193.243	0.0003	90	-0.16412
202	184.243	193.243	0.0003	86	0.14279
203	184.3207	193.3207	0.0031	90	0.11813
203	184.3207	193.3207	0.0031	85	0.15716
203	184.3207	193.3207	0.0031	86	0.37303
203	184.3207	193.3207	0.0031	87	0.40674
203	184.3207	193.3207	0.0031	89	-0.1869

203	184.3207	193.3207	0.0031	91	-0.21818
204	184.3211	193.3211	0.0037	90	0.11269
204	184.3211	193.3211	0.0037	85	-0.15689
204	184.3211	193.3211	0.0037	86	0.37309
204	184.3211	193.3211	0.0037	87	0.40729
204	184.3211	193.3211	0.0037	89	-0.18542
204	184.3211	193.3211	0.0037	91	0.2203
205	184.3216	193.3216	0.0005	85	0.16372
205	184.3216	193.3216	0.0005	86	0.3804
205	184.3216	193.3216	0.0005	87	0.41431
205	184.3216	193.3216	0.0005	89	-0.18954
205	184.3216	193.3216	0.0005	91	-0.22754
205	184.3216	193.3216	0.0005	90	0.11355
206	184.322	193.322	0.0005	85	-0.16333
206	184.322	193.322	0.0005	86	0.38034
206	184.322	193.322	0.0005	87	0.41451
206	184.322	193.322	0.0005	89	-0.18782
206	184.322	193.322	0.0005	91	0.22951
206	184.322	193.322	0.0005	90	0.10768
207	184.3422	193.3422	0.0045	90	-0.11472
207	184.3422	193.3422	0.0045	88	0.15822
207	184.3422	193.3422	0.0045	90	-0.13439
207	184.3422	193.3422	0.0045	86	-0.15742
207	184.3422	193.3422	0.0045	91	-0.17275
207	184.3422	193.3422	0.0045	86	0.49113
207	184.3422	193.3422	0.0045	87	0.2589
207	184.3422	193.3422	0.0045	89	-0.15222
207	184.3422	193.3422	0.0045	91	0.13362
208	184.3428	193.3428	0.0013	88	-0.16078
208	184.3428	193.3428	0.0013	90	-0.14147
208	184.3428	193.3428	0.0013	90	0.10476
208	184.3428	193.3428	0.0013	86	0.49109
208	184.3428	193.3428	0.0013	87	0.25702
208	184.3428	193.3428	0.0013	89	-0.15271
208	184.3428	193.3428	0.0013	91	-0.13064
208	184.3428	193.3428	0.0013	86	0.15698
208	184.3428	193.3428	0.0013	91	-0.16941
209	184.3432	193.3432	0.0001	91	0.127
209	184.3432	193.3432	0.0001	86	0.51185
209	184.3432	193.3432	0.0001	87	0.26643
209	184.3432	193.3432	0.0001	89	-0.15978
209	184.3432	193.3432	0.0001	91	0.17526
209	184.3432	193.3432	0.0001	90	0.12431
209	184.3432	193.3432	0.0001	88	0.14378

209	184.3432	193.3432	0.0001	90	-0.11227
210	184.3436	193.3436	0.0001	86	0.51248
210	184.3436	193.3436	0.0001	87	0.26662
210	184.3436	193.3436	0.0001	89	-0.16104
210	184.3436	193.3436	0.0001	91	-0.17479
210	184.3436	193.3436	0.0001	91	0.12943
210	184.3436	193.3436	0.0001	88	-0.1475
210	184.3436	193.3436	0.0001	90	-0.11566
210	184.3436	193.3436	0.0001	90	-0.13661
211	184.3503	193.3503	0.0001	87	-0.11774
211	184.3503	193.3503	0.0001	89	0.41436
211	184.3503	193.3503	0.0001	91	0.55301
212	184.3518	193.3518	0.0001	87	0.11746
212	184.3518	193.3518	0.0001	89	-0.40996
212	184.3518	193.3518	0.0001	91	0.55629
213	184.3857	193.3857	0.0001	87	-0.12892
213	184.3857	193.3857	0.0001	89	0.43186
213	184.3857	193.3857	0.0001	91	0.50961
213	184.3857	193.3857	0.0001	91	0.1643
214	184.3863	193.3863	0.0002	91	-0.16534
214	184.3863	193.3863	0.0002	87	0.12865
214	184.3863	193.3863	0.0002	89	-0.42776
214	184.3863	193.3863	0.0002	91	0.51296
215	184.408	193.408	0.0159	85	0.26926
215	184.408	193.408	0.0159	87	0.20071
215	184.408	193.408	0.0159	89	0.10791
215	184.408	193.408	0.0159	91	0.15476
215	184.408	193.408	0.0159	86	-0.10168
215	184.408	193.408	0.0159	87	0.50018
215	184.408	193.408	0.0159	89	0.26913
216	184.4087	193.4087	0.0001	80	-0.28329
216	184.4087	193.4087	0.0001	83	-0.13228
216	184.4087	193.4087	0.0001	84	0.59512
216	184.4087	193.4087	0.0001	88	-0.15041
216	184.4087	193.4087	0.0001	90	0.11791
217	184.4087	193.4087	0.0007	87	0.49502
217	184.4087	193.4087	0.0007	89	0.26509
217	184.4087	193.4087	0.0007	85	0.26924
217	184.4087	193.4087	0.0007	87	-0.20224
217	184.4087	193.4087	0.0007	89	-0.10842
217	184.4087	193.4087	0.0007	91	0.1559
218	184.4089	193.4089	0	85	0.22951
218	184.4089	193.4089	0	86	-0.10388
218	184.4089	193.4089	0	87	0.52323

218	184.4089	193.4089	0	89	0.27908
218	184.4089	193.4089	0	91	0.12967
218	184.4089	193.4089	0	85	-0.13845
218	184.4089	193.4089	0	87	-0.10795
219	184.4092	193.4092	0	85	-0.1406
219	184.4092	193.4092	0	87	0.11151
219	184.4092	193.4092	0	85	-0.22839
219	184.4092	193.4092	0	86	-0.10392
219	184.4092	193.4092	0	87	0.52385
219	184.4092	193.4092	0	89	0.27918
219	184.4092	193.4092	0	91	-0.13179
220	184.4098	193.4098	0.0001	80	0.28462
220	184.4098	193.4098	0.0001	83	-0.13263
220	184.4098	193.4098	0.0001	84	0.59988
220	184.4098	193.4098	0.0001	88	-0.15006
220	184.4098	193.4098	0.0001	90	-0.1196
221	184.4426	193.4426	0.003	85	0.29442
221	184.4426	193.4426	0.003	86	-0.1963
221	184.4426	193.4426	0.003	87	0.37331
221	184.4426	193.4426	0.003	89	0.38776
221	184.4426	193.4426	0.003	91	0.19471
222	184.4435	193.4435	0.0035	85	-0.29398
222	184.4435	193.4435	0.0035	86	-0.19603
222	184.4435	193.4435	0.0035	87	0.37309
222	184.4435	193.4435	0.0035	89	0.38693
222	184.4435	193.4435	0.0035	91	-0.19775
223	184.4438	193.4438	0.0003	85	0.30622
223	184.4438	193.4438	0.0003	86	-0.20016
223	184.4438	193.4438	0.0003	87	0.37957
223	184.4438	193.4438	0.0003	89	0.39491
223	184.4438	193.4438	0.0003	91	0.20208
224	184.4448	193.4448	0.0002	85	-0.30636
224	184.4448	193.4448	0.0002	86	-0.19993
224	184.4448	193.4448	0.0002	87	0.37958
224	184.4448	193.4448	0.0002	89	0.39399
224	184.4448	193.4448	0.0002	91	-0.20548
225	184.6588	193.6588	0	89	-0.14013
225	184.6588	193.6588	0	88	-0.34509
225	184.6588	193.6588	0	90	0.42258
225	184.6588	193.6588	0	88	-0.20583
225	184.6588	193.6588	0	90	0.3398
226	184.6597	193.6597	0	88	-0.37357
226	184.6597	193.6597	0	90	0.49773
226	184.6597	193.6597	0	88	0.14569

226	184.6597	193.6597	0	90	-0.2087
226	184.6597	193.6597	0	89	-0.12154
227	184.6601	193.6601	0	89	-0.13536
227	184.6601	193.6601	0	88	-0.20489
227	184.6601	193.6601	0	90	-0.34506
227	184.6601	193.6601	0	88	0.33767
227	184.6601	193.6601	0	90	0.41565
228	184.6611	193.6611	0	88	0.14224
228	184.6611	193.6611	0	90	0.20034
228	184.6611	193.6611	0	88	0.36627
228	184.6611	193.6611	0	90	0.5014
228	184.6611	193.6611	0	89	-0.11887
229	184.6931	193.6931	0.0003	87	-0.10653
229	184.6931	193.6931	0.0003	88	-0.10385
229	184.6931	193.6931	0.0003	90	0.12331
229	184.6931	193.6931	0.0003	88	-0.38561
229	184.6931	193.6931	0.0003	90	0.50191
229	184.6931	193.6931	0.0003	92	-0.13722
230	184.6945	193.6945	0.0008	88	-0.39697
230	184.6945	193.6945	0.0008	90	0.51304
230	184.6945	193.6945	0.0008	92	-0.14162
230	184.6945	193.6945	0.0008	87	-0.10206
231	184.6946	193.6946	0.0003	87	-0.10469
231	184.6946	193.6946	0.0003	88	0.38134
231	184.6946	193.6946	0.0003	90	0.50447
231	184.6946	193.6946	0.0003	92	0.13692
231	184.6946	193.6946	0.0003	88	-0.10326
231	184.6946	193.6946	0.0003	90	-0.12143
232	184.6961	193.6961	0.0008	88	0.39302
232	184.6961	193.6961	0.0008	90	0.51702
232	184.6961	193.6961	0.0008	92	0.1414
232	184.6961	193.6961	0.0008	87	-0.1006
233	184.7013	193.7013	0.0003	82	0.14165
233	184.7013	193.7013	0.0003	85	0.16319
233	184.7013	193.7013	0.0003	86	0.54428
233	184.7013	193.7013	0.0003	87	-0.13292
233	184.7013	193.7013	0.0003	89	0.27348
233	184.7013	193.7013	0.0003	91	-0.22262
234	184.7015	193.7015	0.0004	82	-0.14193
234	184.7015	193.7015	0.0004	85	-0.16316
234	184.7015	193.7015	0.0004	86	0.5439
234	184.7015	193.7015	0.0004	87	-0.13296
234	184.7015	193.7015	0.0004	89	0.27608
234	184.7015	193.7015	0.0004	91	0.22102

235	184.7199	193.7199	0.0024	92	0.43104
235	184.7199	193.7199	0.0024	93	-0.40274
235	184.7199	193.7199	0.0024	92	-0.28238
235	184.7199	193.7199	0.0024	93	0.21758
236	184.7203	193.7203	0.0029	92	0.28027
236	184.7203	193.7203	0.0029	93	0.21986
236	184.7203	193.7203	0.0029	92	0.42755
236	184.7203	193.7203	0.0029	93	0.40663
237	184.7249	193.7249	0.0001	92	0.46917
237	184.7249	193.7249	0.0001	93	-0.42088
237	184.7249	193.7249	0.0001	92	-0.21244
237	184.7249	193.7249	0.0001	93	0.18031
238	184.7253	193.7253	0.0001	92	0.21072
238	184.7253	193.7253	0.0001	93	0.18203
238	184.7253	193.7253	0.0001	92	0.46548
238	184.7253	193.7253	0.0001	93	0.42503
239	184.7856	193.7856	0.0012	83	0.2463
239	184.7856	193.7856	0.0012	84	0.24834
239	184.7856	193.7856	0.0012	88	0.48805
239	184.7856	193.7856	0.0012	90	-0.36177
240	184.7875	193.7875	0.0013	83	0.24579
240	184.7875	193.7875	0.0013	84	0.24732
240	184.7875	193.7875	0.0013	88	0.48578
240	184.7875	193.7875	0.0013	90	0.36579
241	184.8193	193.8193	0.0003	89	-0.26918
241	184.8193	193.8193	0.0003	91	-0.27682
241	184.8193	193.8193	0.0003	89	0.37729
241	184.8193	193.8193	0.0003	91	0.40907
242	184.8202	193.8202	0	89	0.17365
242	184.8202	193.8202	0	91	0.18233
242	184.8202	193.8202	0	87	-0.10496
242	184.8202	193.8202	0	89	0.4289
242	184.8202	193.8202	0	91	0.4571
243	184.8206	193.8206	0.0003	89	-0.37397
243	184.8206	193.8206	0.0003	91	0.41122
243	184.8206	193.8206	0.0003	89	-0.26861
243	184.8206	193.8206	0.0003	91	0.28063
244	184.8215	193.8215	0	87	0.1053
244	184.8215	193.8215	0	89	-0.42743
244	184.8215	193.8215	0	91	0.46265
244	184.8215	193.8215	0	89	0.17117
244	184.8215	193.8215	0	91	-0.18255
245	184.8673	193.8673	0.0009	90	-0.11673
245	184.8673	193.8673	0.0009	87	-0.12637

245	184.8673	193.8673	0.0009	89	0.38792
245	184.8673	193.8673	0.0009	91	0.50813
245	184.8673	193.8673	0.0009	91	-0.15
246	184.8679	193.8679	0.0008	90	0.12191
246	184.8679	193.8679	0.0008	91	0.15087
246	184.8679	193.8679	0.0008	87	0.12659
246	184.8679	193.8679	0.0008	89	-0.38434
246	184.8679	193.8679	0.0008	91	0.51188
247	184.8686	193.8686	0.0001	87	-0.1295
247	184.8686	193.8686	0.0001	89	0.3954
247	184.8686	193.8686	0.0001	91	0.52805
247	184.8686	193.8686	0.0001	90	-0.11213
248	184.8691	193.8691	0	87	0.12973
248	184.8691	193.8691	0	89	-0.39168
248	184.8691	193.8691	0	91	0.53161
248	184.8691	193.8691	0	90	0.11725
249	184.9039	193.9039	0.0019	88	-0.11766
249	184.9039	193.9039	0.0019	92	0.55919
249	184.9039	193.9039	0.0019	93	-0.39852
250	184.9041	193.9041	0.0021	88	-0.11772
250	184.9041	193.9041	0.0021	92	0.55564
250	184.9041	193.9041	0.0021	93	0.40324
251	184.9811	193.9811	0.0003	85	-0.15901
251	184.9811	193.9811	0.0003	86	0.34836
251	184.9811	193.9811	0.0003	87	0.50857
251	184.9811	193.9811	0.0003	89	-0.20158
251	184.9811	193.9811	0.0003	91	0.20673
252	184.9815	193.9815	0.0004	85	0.15905
252	184.9815	193.9815	0.0004	86	0.34905
252	184.9815	193.9815	0.0004	87	0.50804
252	184.9815	193.9815	0.0004	89	-0.20337
252	184.9815	193.9815	0.0004	91	-0.20506

Supporting Information References

(1) (a) Hawthorne, M. F.; Young, D. C.; Andrews, T. D.; Howe, D. V.; Pilling, R. L.; Pitts, A. D.; Reintjes, M.; Warren, L. F., Jr.; Wegner, P. A. *J. Am. Chem. Soc.* **1968**, *90*, 879-896. (b) Hawthorne, M. F.; Young, D. C.; Garrett, P. M.; Owen, D. A.; Schwerin, S. G.; Tebbe, F. N.; Wegner, P. A. *J. Am. Chem. Soc.* **1968**, *90*, 862-868.

(2) Gaussian 09, Revision B.01, Frisch, M.J.; Trucks, G.W.; Schlegel, H.B.; Scuseria, G.E.; Robb, M.A.; Cheeseman, J.R.; Scalmani, G.; Barone, V.; Mennucci, B.; Petersson, G.A.; Nakatsuji, H.; Caricato, M.; Li,X.; Hratchian, H.P.; Izmaylov, A.F.; Bloino, J.; Zheng, G.; Sonnenberg, J.L.; Hada, M.; Ehara, M.; Toyota, K.; Fukuda, R.; Hasegawa, J.; Ishida, M.; Nakajima, T.; Honda, Y.; Kitao, O.; Nakai, H.; Vreven, T.; Montgomery, J.A., Jr.; Peralta, J.E.; Ogliaro, F.; Bearpark, M.; Heyd, J.J.; Brothers, E.; Kudin, K.N.; Staroverov, V.N.; Kobayashi, R.; Normand, J.; Raghavachari, K.; Rendell, A.; Burant, J.C.; Iyengar, S.S.; Tomasi, J.; Cossi, M.; Rega, N.; Millam, N.J.; Klene, M.; Knox, J.E.; Cross, J.B.; Bakken, V.; Adamo, C.; Jaramillo, J.; Gomperts, R.; Stratmann, R.E.; Yazyev, O.; Austin, A.J.; Cammi, R.; Pomelli, C.; Ochterski, J. W.; Martin, R.L.; Morokuma, K.; Zakrzewski, V.G.; Voth, G.A.; Salvador, P.; Dannenberg, J.J.; Dapprich, S.; Daniels, A.D.; Farkas, Ö.; Foresman, J.B.; Ortiz, J.V.; Cioslowski, J.; Fox, D.J. Gaussian, Inc., Wallingford CT, 2009.

(3) (a) Becke, A. D. *J. Chem. Phys.* **1993**, *98*, 5648-5652. (b) Grimme, S.; Antony, J.; Ehrlich, S.; Krieg, H. *J. Chem. Phys.* **2010**, *132*, 154104/154101-154104/154119.

(4) (a) Hay, P. J.; Wadt, W. R. *J. Chem. Phys.* **1985**, *82*, 299-310. (b) Ehlers, A. W.; Boehme, M.; Dapprich, S.; Gobbi, A.; Hoellwarth, A.; Jonas, V.; Koehler, K. F.; Stegmann, R.; Veldkamp, A. *Chem. Phys. Lett.* **1993**, *208*, 111-114. (c) Roy, L. E.; Hay, P. J.; Martin, R. L. *J. Chem. Theory Comput.* **2008**, *4*, 1029-1031.

(5) Ditchfield, R.; Hehre, W. J.; Pople, J. A. *J. Chem. Phys.* **1971**, *54*, 724-728.

(6) Mulliken, R. S. *J. Chem. Phys.* **1955**, *23*, 1833-1840.
